# Assessing spatial and temporal transferability of cycleway impact models for bike-share usage in London

**DOI:** 10.1038/s41598-026-52211-5

**Published:** 2026-05-20

**Authors:** Yuan Ma, Yasir Ali, Craig Morton, He Haitao

**Affiliations:** School of Architecture, Building, and Civil Engineering, Loughborough University, Leicestershire, LE11 3TU UK

**Keywords:** Cycleway, Cycling, Bike-share, Transferability, Quasi-experimental methods, Impact evaluation, Time series models, Contextual calibration, Mathematics and computing, Social sciences

## Abstract

Assessing spatial and temporal transferability in models of cycleway impacts can inform evidence-based transport policies and wider implementation of cycling infrastructure. Yet, limited research on model transferability constrains their practical application in infrastructure planning. This study examines spatial and temporal transferability of models estimating the effects of cycleway implementation on bike-share usage. Using 13 years of fortnightly data from London’s bike-share scheme across Cycleways 1, 3, and 6 (over 14,000 time-station observations), we combine three modelling frameworks (Autoregressive Integrated Moving Average with Exogenous variables [ARIMAX], Generalised Additive Models [GAM], and Generalised Additive Mixed Models with ARMA errors [GAMM+ARMA]) with three transferability strategies (direct transferability, contextual calibration, and local re-estimation). Results show that contextual calibration generally outperforms the other two strategies, reducing errors by 20–80% compared with direct transferability and by 5–45% compared with local re-estimation. Cycleway interventions are positively associated with bike-share usage, whilst demographic covariates exhibit spatial heterogeneity. These findings highlight the value of partial model adaptation for balancing transferability with local relevance and suggest contextual calibration as a practical strategy for transferable cycleway intervention modelling. This study provides insights for transport authorities to prioritise investment, scale cycling infrastructure efficiently, and adapt successful interventions to diverse urban contexts.

## Introduction

Cycling is increasingly recognised as a key active transport mode, supporting sustainable, healthy, and economically efficient urban mobility^1^. To promote cycling, cities are investing in connected and well-designed cycling networks to enhance safety and ridership, alongside implementing bike-share systems to enhance accessibility and convenience^2,3^. Empirical studies^3^ suggest that users particularly prefer cycling infrastructures that are physically protected, sufficiently wide, and clearly signposted. As such, dedicated cycleways are deemed promising for future development of urban cycling infrastructure^4^. Whilst dedicated cycleways provide clear advantages over conventional cycling facilities, their development can be costly, particularly when involving substantial infrastructure, such as bridges or tunnels. A clearer understanding of their impacts on cycling activity is, therefore, essential to help inform future planning and investment decisions^5^.

Several modelling frameworks have been employed to assess the impact of cycling infrastructure on cycling, typically focussing on context-specific effects. The difference-in-differences design is widely used, often combined with statistical models^5,6^. For example, a previous study^7^ using a fixed effects Poisson panel model showed a positive association between new infrastructure and cycling activity. Discrete choice models, including mixed multinomial logit^8,9^ and binary logit models^5^, have also been used to capture preference heterogeneity, suggesting that cycling infrastructure promotes cycling uptake. Time series and semi-parametric models provide flexibility in capturing temporal autocorrelation and non-linear time trends. For instance, generalised additive models and generalised additive mixed models have been used to examine non-linear and spatiotemporal variations in bike-share usage following changes in cycling infrastructure density^10^. In addition, cycleway impacts have been studied for other micromobility modes, such as e-scooters^11,12^ and e-bikes^13^. Although these studies have advanced understanding of how cyclists respond to infrastructure interventions, they have primarily focused on single spatial or temporal contexts, leaving the transferability of cycleway intervention models largely unexplored.

Transferability refers to the extent to which a model developed in one context remains valid in another similar context^14^ and is typically examined in spatial and temporal domains^15^. Numerous studies^14–18^ have evaluated transferability in travel behaviour modelling, particularly in relation to cycling mode choice. For instance, Santoso and Tsunokawa^16^ compared updating techniques to improve the spatial transferability of work-trip mode choice models in developing countries. Similarly, the temporal transferability of home-based work choice models in Toronto has been assessed^17^, whilst spatial transferability across Finnish cities has also been examined^18^. More recently, transfer learning techniques have been introduced to improve cross-context prediction in multimodal transport systems. For instance, machine learning models trained on bike-share and public transit data in one city have been effectively adapted to others through fine-tuning, without requiring full retraining^19^. Existing studies on cycling demand analysis have mainly focussed on forecasting aggregate demand or mode share. For example, spatial transferability of direct-demand models for cyclist volumes^20^ has been evaluated, with findings indicating substantial reductions in predictive accuracy when models were applied directly outside their original areas. Despite prior investigations^14–20^, no research exists on assessing the impact of dedicated cycleway implementation on cycling behaviour. Thus, it remains unclear whether a model developed on one cycleway can successfully estimate intervention effects on a different cycleway, or whether a model developed for an initial segment of a cycleway can be effectively transferred to an extension. Addressing this gap is critical for helping policymakers evaluate and adapt cycling infrastructure strategies over time, based on transferable insights from earlier interventions.

To fill this gap, the objective of this study is to evaluate the spatial and temporal transferability of intervention models for estimating the impact of cycleway implementation on bike-share usage. Specifically, this study answers two research questions: (i) How do different modelling approaches (Autoregressive Integrated Moving Average with Exogenous variables, Generalised Additive Models, and Generalised Additive Mixed Models) perform in varying spatial and temporal contexts? (ii) Which model transferability strategy (direct transferability, contextual calibration, and local re-estimation) provides the lowest error across spatial and temporal contexts? By answering these research questions, this study contributes to the theoretical and practical understanding of cycling infrastructure interventions. Theoretically, to the best of the authors’ knowledge, this study presents the first systematic and rigorous investigation of the spatial and temporal transferability of intervention impact models for cycling infrastructure. Through the comparison of three modelling approaches under three transferability strategies, the study provides new insights into the transferability of cycleway intervention models. Practically, the results suggest that contextual calibration can reuse the source model by initialising parameters in new spatial and temporal settings, without requiring full model retraining. Compared with full re-estimation, contextual calibration is conceptually aligned with approaches in the transfer learning literature^21,22^ that are evaluated under limited target-data settings, where the available observations are fewer than in the source domain. These transfer learning studies evaluate predictive accuracy under varying target data availability and report that transferring or fine-tuning models can often mitigate performance degradation relative to training from scratch, although the magnitude and direction of accuracy trade-offs are context dependent^23,24^.

## Results

### Spatial transferability

In spatial transferability analysis, across all models and cycleways, the contextual calibration strategy yields more favourable model diagnostics compared to direct transferability and local re-estimation, as indicated by lower values of the Akaike Information Criterion (AIC), which balances model goodness-of-fit and complexity, and higher adjusted or pseudo-*R²* scores (Tables 1, 2, 3). Contextual calibration also maintains directionality and significance for key intervention variables, indicating greater cross-context generalisability. All inferences are made assuming a 95% confidence level.

In the ARIMAX models (Table 1), the *time-since-intervention* variable is statistically significant and negative across all transferability strategies, suggesting a sustained decline in bike-share usage after the cycleway intervention. In contrast, the *post-intervention indicator* is consistently non-significant. Although the impact of cycleway implementation appears to be negative, the contextual calibration models achieve better spatial transferability, as reflected in lower AIC values and slightly higher pseudo-*R²*, indicating superior overall model performance. Demographic parameters exhibit cycleway-specific associations as well. For instance, the *distance to work (between 2 and 5 km)* is negatively associated with bike-share usage under direct transferability but shifts to a positive association under contextual calibration for both cycleways. Other covariates, such as the *proportion of females*, *employment status*, and *rental housing* also vary in direction and significance across strategies and cycleways. Notably, contextual calibration and local re-estimation produce similar directional effects for some demographic variables, in contrast to the direct transferability strategy, which shows variability in direction across cycleways.

**Table 1:** Results of the ARIMAX models under different transferability strategies for spatial transferability.

Transferability strategies	Cycleway 1			Cycleway 3		
	DT	CC	LRE	DT	CC	LRE
	Estimate (S.E.)	Estimate (S.E.)	Estimate (S.E.)	Estimate (S.E.)	Estimate (S.E.)	Estimate (S.E.)
Intercept	5.3896 (0.7534)	–	5.0596 (0.3208)	5.3896 (0.7534)	–	6.1401 (0.2704)
Post-intervention indicator	–	–	–	–	–	–
Time-since-intervention	− 0.0154 (0.0012)	− 0.0126 (0.0003)	− 0.0211 (0.0013)	− 0.0154 (0.0012)	− 0.0125 (0.0002)	− 0.0208 (0.0013)
Method to travel to work (bicycle)	–	− 0.6398 (0.0586)	− 0.1334 (0.0181)	–	–	–
Distance to work less than 2 km	–	− 0.0548 (0.0119)	–	–	0.0650 (0.0083)	–
Distance to work between 2–5 km	− 0.0697 (0.0144)	0.2471 (0.0341)	− 0.0336 (0.0078)	− 0.0697 (0.0144)	0.0937 (0.0129)	0.0651 (0.0059)
Female	–	− 0.0165 (0.0063)	–	–	–	− 0.0235 (0.0030)
Age between 20–49	–	0.0987 (0.0146)	–	–	− 0.0171 (0.0034)	–
Age between 50–74	–	0.1448 (0.0173)	–	–	− 0.0438 (0.0062)	–
Employed	–	− 0.1130 (0.0145)	–	–	0.0219 (0.0026)	–
Rented	0.0213 (0.0034)	–	–	0.0213 (0.0034)	− 0.0135 (0.0018)	− 0.0084 (0.0018)
Model statistics						
Log-likelihood	–	− 8542.64	− 8573.42	–	− 7413.04	− 7415.77
Akaike information criterion	–	17105.27	17166.85	–	14846.07	14851.55
Pseudo *R*-squared	–	0.17	0.16	–	0.16	0.16
Number of observations	4976	4976	4976	4680	4680	4680

“-” not statistically significant or not included. Likelihood-based goodness-of-fit measures (AIC, log-likelihood, and R²) are not reported for the direct transferability strategy because model parameters are not estimated on the target data. These statistics are defined conditional on parameter estimation and are therefore not directly meaningful or comparable when all parameters are fixed and transferred from the source model to the target context. In addition, the performance of the direct transferability strategy is evaluated using predictive accuracy measures in the subsequent analysis. Table 5 reports RMSE and MAE for all transferability strategies, including direct transferability, across all modelling frameworks. Because these metrics are computed on the target data and do not rely on parameter re-estimation, they provide a consistent and comparable basis for assessing performance across strategies.

In the GAM models (Table 2), the *post-intervention indicator* and *time-since-intervention* variables are positive and statistically significant across all strategies and cycleways. This relationship suggests an immediate and sustained increase in bike-share usage following the cycleway implementation. Similar to the ARIMAX models, demographic covariates exhibit variation in magnitude and direction across cycleways and transferability strategies as well. For instance, the *proportion of individuals who cycle to work* is positively associated with bike-share usage under the direct transferability strategy, but this relationship becomes negative under contextual calibration and local re-estimation. In contrast, the *commuting distance of 2–5 km* is negatively associated with bike-share usage under direct transferability, but this association turns positive under the contextual calibration model, particularly in Cycleway 1. The *proportion of female residents* shows a negative relationship with bike-share usage across all strategies and cycleways. *Rental tenure* is positively associated under direct transferability but shifts to a negative relationship under contextual calibration and local re-estimation strategies in both cycleways. Additionally, some key demographic variables maintain same direction under contextual calibration and local re-estimation.

**Table 2:** Results of the GAM model under different transferability strategies for spatial transferability.

Transferability strategies	Cycleway 1			Cycleway 3		
	DT	CC	LRE	DT	CC	LRE
	Estimate (S.E.)	Estimate (S.E.)	Estimate (S.E.)	Estimate (S.E.)	Estimate (S.E.)	Estimate (S.E.)
Intercept	2.0654 (0.0852)	14.1878 (0.0776)	3.4740 (0.0657)	2.0654 (0.0852)	14.8870 (0.0749)	2.4420 (0.0620)
Post-intervention indicator	0.3351 (0.0065)	0.1875 (0.0051)	0.1877 (0.0051)	0.3351 (0.0065)	0.5696 (0.0048)	0.4823 (0.0049)
Time-since-intervention	0.0587 (0.0011)	0.0656 (0.0009)	0.0657 (0.0009)	0.0587 (0.0011)	0.0412 (0.0008)	0.0673 (0.0009)
Method to travel to work (bicycle)	0.0166 (0.0004)	− 0.8533 (0.0029)	− 0.1183 (0.0006)	0.0166 (0.0004)	− 0.0069 (0.0006)	− 0.0150 (0.0005)
Distance to work less than 2 km	0.0058 (0.0004)	0.0945 (0.0005)	–	0.0058 (0.0004)	− 0.0528 (0.0004)	− 0.0090 (0.0003)
Distance to work between 2–5 km	− 0.0851 (0.0006)	0.4864 (0.0017)	0.0198 (0.0003)	− 0.0851 (0.0006)	− 0.1127 (0.0005)	–
Female	− 0.0154 (0.0005)	− 0.1631 (0.0005)	− 0.0335 (0.0002)	− 0.0154 (0.0005)	− 0.0284 (0.0002)	− 0.0042 (0.0001)
Age between 20–49	0.0008 (0.0001)	0.0843 (0.0005)	–	0.0008 (0.0001)	− 0.1110 (0.0003)	–
Age between 50–74	− 0.0049 (0.0002)	0.0906 (0.0006)	–	− 0.0049 (0.0002)	− 0.1006 (0.0004)	–
Employed	− 0.0084 (0.0001)	− 0.1311 (0.0005)	− 0.0020 (0.0001)	− 0.0084 (0.0001)	0.0633 (0.0002)	–
Rented	0.0245 (0.0001)	− 0.0856 (0.0003)	–	0.0245 (0.0001)	− 0.0419 (0.0001)	− 0.0077 (0.0001)
Model statistics						
Log-likelihood	–	− 486817.9	− 532725.1	–	− 434580.9	− 557501.1
Akaike information criterion	–	973675.7	1,065,482	–	869201.7	1,115,034
Adjusted *R*-squared	–	0.27	0.19	–	0.33	0.12
Number of observations	4976	4976	4976	4680	4680	4680

In the GAMM+ARMA models (Table 3), the *post-intervention indicator* and the *time-since-intervention* variables remain statistically significant and positive across all transferability strategies and cycleways. As with the GAM models, the contextual calibration strategy shows superior model performance as well. For instance, in Cycleway 3, the contextual calibration model achieves an adjusted *R*² of 0.33, compared to 0.11 under local re-estimation. Compared to the GAM model, incorporating temporal autocorrelation slightly amplifies the estimated effects of demographic variables, such as *commuting distance*. The contextual calibration strategy maintains strong explanatory performance with this complex modelling framework, underscoring its robustness in spatial transferability even when temporal dependencies are present. Whilst demographic covariates continue to exhibit spatial heterogeneity, the direction of key variables, such as *commuting distance*, *gender*, *rental tenure*, and *employment status*, remains largely consistent between contextual calibration and local re-estimation. Overall, these findings suggest the value of the GAMM+ARMA framework in capturing time-dependent patterns whilst preserving the explanatory coherence and transferability advantages provided by the contextual calibration strategy.

**Table 3:** Results of the GAMM+ARMA model under different transferability strategies for spatial transferability.

Transferability strategies	Cycleway 1			Cycleway 3		
	DT	CC	LRE	DT	CC	LRE
	Estimate (S.E.)	Estimate (S.E.)	Estimate (S.E.)	Estimate (S.E.)	Estimate (S.E.)	Estimate (S.E.)
Intercept	0.7320 (0.1822)	15.1017 (0.1675)	3.4022 (0.1338)	0.7320 (0.1822)	14.8123 (0.1328)	2.0043 (0.1270)
Post-intervention indicator	0.2382 (0.0091)	0.0918 (0.0074)	0.2335 (0.0074)	0.2382 (0.0091)	0.4615 (0.0070)	0.3234 (0.0073)
Time-since-intervention	0.0778 (0.0021)	0.0546 (0.0019)	0.0661 (0.0018)	0.0778 (0.0021)	0.0431 (0.0015)	0.0749 (0.0017)
Method to travel to work (bicycle)	0.0249 (0.0019)	− 0.8550 (0.0070)	− 0.1184 (0.0013)	0.0249 (0.0019)	− 0.0070 (0.0011)	− 0.0151 (0.0012)
Distance to work less than 2 km	0.0090 (0.0020)	0.0947 (0.0014)	–	0.0090 (0.0020)	− 0.0528 (0.0007)	− 0.0092 (0.0005)
Distance to work between 2–5 km	− 0.0861 (0.0025)	0.4874 (0.0041)	0.0199 (0.0005)	− 0.0861 (0.0025)	− 0.1124 (0.0010)	–
Female	− 0.0156 (0.0021)	− 0.1633 (0.0012)	− 0.0336 (0.0004)	− 0.0156 (0.0021)	− 0.0283 (0.0003)	− 0.0042 (0.0002)
Age between 20–49	0.0017 (0.0008)	0.0844 (0.0012)	–	0.0017 (0.0008)	− 0.1109 (0.0006)	–
Age between 50–74	− 0.0024 (0.0010)	0.090 (0.0015)	–	− 0.0024 (0.0010)	− 0.1005 (0.0006)	–
Employed	− 0.0099 (0.0005)	− 0.1312 (0.0014)	− 0.0021 (0.0001)	− 0.0099 (0.0005)	0.0633 (0.0003)	–
Rented	0.0229 (0.0005)	− 0.0857 (0.0007)	–	0.0229 (0.0005)	− 0.0418 (0.0002)	− 0.0078 (0.0001)
Model statistics						
Log-likelihood	–	− 173735.9	− 189719.7	–	− 209298.6	− 226524.4
Akaike Information Criterion	–	347499.8	379459.4	–	418625.3	453070.9
Adjusted *R*-squared	–	0.26	0.19	–	0.33	0.11
Number of observations	4976	4976	4976	4680	4680	4680

### Temporal transferability

Across all three modelling frameworks, the contextual calibration strategy suggests strong and stable performance (Table 4). In the ARIMAX model, contextual calibration achieves a notably lower AIC than local re-estimation. In the GAM, contextual calibration and local re-estimation exhibit similar AIC values, but contextual calibration yields slightly higher adjusted *R*², indicating improved explanatory power. The advantage of contextual calibration becomes more distinct in GAMM+ARMA, where it achieves a much lower AIC (26652.86) compared to local re-estimation.

**Table 4:** Results of the three modelling frameworks under different transferability strategies for temporal transferability.

Transferability strategies	ARIMAX			GAM			GAMM+ARMA		
	DT	CC	LRE	DT	CC	LRE	DT	CC	LRE
	Estimate	Estimate	Estimate	Estimate	Estimate	Estimate	Estimate	Estimate	Estimate
Intercept	4.9433 (0.1851)	–	5.5270 (0.1022)	5.0199 (0.0367)	3.5796 (0.0760)	3.5778 (0.0760)	5.0419 (0.0408)	3.5380 (0.0759)	3.5782 (0.0685)
Post-intervention indicator	0.5071 (0.1133)	− 0.0079 (0.0020)	–	0.0791 (0.0106)	0.3163 (0.0106)	0.3165 (0.0106)	0.0940 (0.0094)	0.3139 (0.0095)	0.3156 (0.0090)
Time-since-intervention	–	–	–	0.0896 (0.0074)	0.2314 (0.0086)	0.2315 (0.0086)	0.0894 (0.0072)	0.2389 (0.0082)	0.2334 (0.0073)
Method to travel to work (bicycle)	0.0564 (0.0117)	− 0.0321 (0.0058)	–	0.02280 (0.0003)	− 0.0012 (0.0003)	–	0.0200 (0.0011)	− 0.0069 (0.0014)	–
Distance to work less than 2 km	–	0.0097 (0.0033)	0.0122 (0.0031)	0.0120 (0.0002)	0.0102 (0.0002)	0.0103 (0.0002)	0.0116 (0.0006)	0.0072 (0.0008)	0.0067 (0.0007)
Distance to work between 2–5 km	− 0.1334 (0.0080)	− 0.0717 (0.0038)	− 0.0790 (0.0040)	− 0.0916 (0.0002)	− 0.0707 (0.0003)	− 0.0708 (0.0002)	− 0.0889 (0.0008)	− 0.0681 (0.0010)	− 0.0682 (0.0009)
Rented	0.0249 (0.0033)	0.0259 (0.0009)	0.0172 (0.0017)	0.0193 (0.0001)	0.0153 (0.0001)	0.0152 (0.0001)	0.0188 (0.0003)	0.0156 (0.0004)	0.0153 (0.0004)
Model statistics									
Log-likelihood	–	− 1856.87	− 1997.3	–	− 76551.47	− 76556.68	–	− 13314.43	− 13705.16
Akaike Information Criterion	–	3727.74	4004.59	–	153134.9	153143.4	–	26652.86	27430.32
Adjusted *R*-squared	–	0.15	0.18	–	0.41	0.40	–	0.41	0.41
Number of observations	1888	1888	1888	1888	1888	1888	1888	1888	1888

For the effect of cycleway implementation on bike-share usage, in the ARIMAX framework, direct transferability strategy produces a statistically significant and positive estimate for the *post-intervention indicator* (0.5071), suggesting an immediate increase in usage. In contrast, contextual calibration yields a small but negative estimate, as partial parameter initialisation in the ARIMAX framework can shift the intervention effect when local autocorrelation patterns differ from the source, and the effect is not retained under local re-estimation. *Time-since-intervention* is not significant from all ARIMAX specifications. By comparison, GAM and GAMM+ARMA indicate significant and positive *post-intervention* and *time-since-intervention* effects across all strategies, suggesting immediate increase and sustained growth in usage over time.

The estimated effects of demographic parameters remain largely stable across time. For example, the *distance to work (less than 2 km)* and *rental tenure* consistently shows positive associations with bike-share usage across all models and strategies, whilst the *distance to work (between 2 and 5 km)* is negative. Notably, as the temporal transferability occurs in the same cycleway over time, demographic parameters show greater directional consistency across all three strategies, especially in the GAM and GAMM+ARMA models. This finding contrasts with the spatial transferability results, where spatial heterogeneity across cycleways led to greater variation in the direction of covariate effects.

As a robustness check, we conducted a placebo test by assigning a fake intervention date (March 2018) to the Cycleway 6 extension, before its actual opening in September 2018. The placebo models produced weaker and directionally inconsistent intervention effects and substantially higher predictive error, indicating that the temporal transferability results are robust and not driven by spurious temporal trends.

Overall, these findings suggest that cycleway intervention models can be successfully transferred across temporal contexts by appropriate calibration. Contextual calibration is a practical and efficient strategy for temporal transferability, striking a balance between model flexibility and structural continuity, whilst improving the reliability in assessing dynamic cycling behaviour. To the authors’ best knowledge, occasional coincident shocks, such as public transport disruptions (e.g., Tube strikes) or pricing changes in the bike-share system, occurred during the study period. However, there is no indication that they had a significant or systematic influence on the transferability of the cycleway intervention models.

### Model performance comparison

Table 5 presents the predictive performance of transferability strategies across different modelling frameworks using Root Mean Square Error (RMSE) and Mean Absolute Error (MAE) in spatial (Cycleway 1 and 3) and temporal (Cycleway 6 extension) contexts. To ensure comparability, ARIMAX predictions (fitted on log-transformed outcomes) were back-transformed to the original scale before evaluation, and error metrics (RMSE and MAE) were calculated on the original scale of bike-share trips across all models. Across all three modelling frameworks, the contextual calibration strategy yields the lowest errors, with the only exception observed in the ARIMAX framework for temporal transferability (Cycleway 6 extension). RMSE and MAE values under temporal transferability are lower than those under spatial transferability across most modelling frameworks and strategies, indicating more accurate predictions in temporal transferability contexts.

Contextual calibration regularly outperforms direct transferability and local re-estimation particularly in GAM and GAMM+ARMA frameworks. Whilst local re-estimation occasionally produces slightly lower errors than contextual calibration, for instance, RMSE of 229.68 (local re-estimation) and 231.11 (contextual calibration) in ARIMAX for Cycleway 6 extension, the difference is minimal. Importantly, contextual calibration generally achieves this level of accuracy with fewer adjustments and greater efficiency. In contrast, direct transferability consistently exhibits the highest RMSE and MAE values, showing limited adaptability without contextual adaptation. These results complement earlier findings, further reinforcing that the contextual calibration provides a robust and balanced strategy to cycleway intervention model transferability. By partially adapting model parameters whilst maintaining core structure, the contextual calibration shows interpretability and prediction accuracy across spatial and temporal contexts. Overall, these results suggest that contextual calibration is a practical and robust strategy for model transferability in evaluating cycling infrastructure interventions.

**Table 5:** RMSE and MAE of three modelling frameworks across transferability strategies and contexts.

Cycleway #	ARIMAX			GAM			GAMM+ARMA		
	DT	CC	LRE	DT	CC	LRE	DT	CC	LRE
1 (RMSE)	482.75	324.22	366.63	2003.88	317.58	452.67	1772.65	317.61	451.68
3 (RMSE)	650.46	379.23	395.98	739.50	368.47	691.31	743.92	367.83	692.58
6_E (RMSE)	303.10	231.11	229.68	961.83	184.16	227.79	921.31	185.11	229.66
1 (MAE)	351.47	268.69	274.80	1025.22	251.23	408.22	940.06	250.71	406.22
3 (MAE)	497.37	286.41	294.81	585.70	275.51	535.77	591.77	275.51	536.38
6_E (MAE)	227.09	184.06	182.25	680.72	145.93	169.27	656.48	147.29	171.18

### Effects of census data on transferability analysis

This study initially used 2021 Census data, whereas the analysis period consists of data from 2012, which is closer to the 2011 Census, thus raising questions about Census selection and its consequent effects. To demonstrate the robustness of findings and the developed models, we conducted an additional analysis incorporating the 2011 and 2021 UK Census datasets. Specifically, demographic variables from the 2011 Census were assigned to the period 2012–2018, and those from the 2021 Census were assigned to 2019–2024. All ARIMAX, GAM, and GAMM+ARMA models were re-estimated for spatial and temporal transferability contexts using this temporally matched census assignment (see Tables 7, 8, 9, 10, 11 in the appendix). This new analysis supports the conclusions drawn using 2021 Census data. Specifically, the estimated cycleway intervention effects remain consistent in direction and statistical significance across modelling frameworks and transferability contexts. Notably, the contrasting patterns between ARIMAX and GAM-based models are maintained, with GAM and GAMM+ARMA indicating positive and sustained intervention effects, whilst ARIMAX results reflect its more restrictive linear temporal structure. Importantly, the relative performance of transferability strategies is also stable, implying that contextual calibration continues to show the strongest overall performance compared with direct transferability and local re-estimation across spatial and temporal transferability.

In the additional analysis, although some variation is observed in the estimated coefficients of the “method of travel to work (bicycle)” variable across the spatial transferability context, particularly for Cycleway 1, this variability reflects spatial heterogeneity in local commuting features rather than instability in the modelling framework. As an area-level demographic indicator, this variable captures contextual differences in pre-existing cycling behaviour across cycleways, which are expected to vary when models are transferred between distinct spatial contexts. Importantly, under temporal transferability in the additional analysis, the estimated relationship of this variable is more stable. Across all specifications, these variations do not significantly affect the estimated cycleway intervention effects or the relative performance of transferability strategies, and therefore do not influence the main conclusions of the study.

Building on these observations, in the spatial transferability context, some demographic coefficients vary in magnitude and occasionally in direction when using temporally matched census data, reflecting differences between the 2011 and 2021 census baselines. We also observe a minor change in the relative ranking of predictive errors between direct transferability and local re-estimation for one spatial transferability case (Cycleway 1). However, these differences are modest, neither affect the interpretation of intervention effects nor change the main conclusion that contextual calibration offers a robust and balanced transferability strategy. Results for Cycleway 3 and the temporal transferability (Cycleway 6 extension) remain consistent with those reported earlier.

### Effects of the buffer around the cycleway on transferability

This study used a 300 m buffer around the cycleway for transferability analyses based on an earlier study^25^. However, this threshold is arbitrary and does not provide a theoretical justification. Thus, we conducted a sensitivity analysis using alternative buffer distances of 200 m and 400 m. This analysis includes all three modelling frameworks (ARIMAX, GAM, and GAMM+ARMA), all transferability strategies (direct transferability, contextual calibration, and local re-estimation), and both spatial and temporal transferability contexts. Note that the detailed results are presented in Tables 12, 13, 14, 15, 16, 17, 18, 19, 20, 21 in the appendix.

Across alternative buffer distances (200 m and 400 m), the results are highly consistent with those reported when using a 300 m buffer. In particular, the direction and statistical significance of intervention-related effects (post-intervention indicator and time-since-intervention) remain stable, and the relative performance patterns across transferability strategies and modelling frameworks are unchanged. Whilst coefficient magnitudes and sample sizes vary with buffer size, these changes do not affect the main conclusions of this study. These findings indicate that the main results are not sensitive to variations in the catchment area definition. In particular, the 300 m buffer reflects the spatial relationship between cycleways and nearby bike-share stations, capturing stations located in a short and reasonable distance of cycling infrastructure whilst limiting the inclusion of more distant stations that are unlikely to be directly influenced. As a result, the estimated intervention effects and transferability patterns observed at 300 m are stable and representative, rather than driven by an arbitrary buffer choice.

Note that we did not extend the sensitivity analysis to a 500 m buffer, which is likely to capture areas that are not directly influenced by the cycleway intervention, particularly in dense urban environments. Expanding the buffer to this scale would increase the risk of incorporating unrelated stations and contextual characteristics, thereby affecting the estimated intervention effects and interpretability. Accordingly, the analysis is restricted to buffer sizes that reflect the spatial influence of cycleway implementation and support a clear evaluation of intervention effects and model transferability.

## Discussion

Across GAM and GAMM+ARMA modelling frameworks and transferability strategies, the estimated effects of cycleway implementation on bike-share usage are positive. Some ARIMAX specifications, however, suggest negative post-intervention trends compared with GAM-based models. This finding likely reflects ARIMAX’s reliance on a linear time trend, which can misrepresent rapid growth followed by stabilisation as an overall decline. In contrast, GAM–based models flexibly capture non-linear temporal dynamics and show sustained increases. These results indicate that cycleway interventions tend to increase bike-share use, consistent with prior findings that dedicated bicycle pathways can promote cycling due to improved safety and comfort^26^.

Observed inconsistencies in demographic coefficients primarily reflect spatial heterogeneity rather than modelling instability. Cycleways 1, 3, and 6 serve different areas with distinct demographic and land use characteristics. These features can influence how particular demographic groups engage with bike-share usage^27^. As such, demographic variables, such as gender proportion, rental tenure, commuting distance, and employment status, may not represent stable or consistent bike-share behaviour across corridors. This pattern is more obvious in the spatial transferability, where demographic effects vary more strongly than in the temporal transferability. Spatial transferability requires models to generalise across cycleways with distinct demographic features and built environments, whereas temporal transferability primarily tests model robustness over time in a largely unchanged spatial context. Thus, these inconsistencies highlight context dependence in the interpretation of the relationship between demographic features and cycling behaviour, rather than unexplained noise. Importantly, this variation in demographic coefficients should not be interpreted as model instability. Model stability should not be interpreted as directional consistency of all covariate coefficients across spatial and temporal transferability contexts. Instead, stability should be assessed via key intervention effects and overall model performance. As shown consistently across GAM and GAMM+ARMA frameworks, the intervention variables (post-intervention indicator and time-since-intervention) are stable (in sign and significance) across transferability contexts and strategies. Some demographic covariates show greater variability, especially under direct transferability, which can be attributed to insufficient local adaptation. In direct transferability, the model applies parameters estimated for Cycleway 6 to Cycleways 1 and 3 without adjustment, resulting in greater directional inconsistency in demographic covariate effects. In contrast, contextual calibration partially updates model parameters to the target cycleway, yielding more stable and interpretable demographic effects. This pattern indicates that the inconsistencies are methodological outcomes of transferability, not the structural weaknesses of modelling frameworks or transferability strategies.

These inconsistencies also suggest that demographic variables are not directly transferable factors across contexts. These findings do not suggest that demographic features are not important but rather highlight that the influence of demographic factors on cycling behaviour is context-dependent. Our study highlights that whilst demographic associations vary spatially, the estimated impacts of cycleway interventions are robust and transferable across spatial and temporal contexts. This finding implies that policymakers can generalise conclusions about the impact of cycleway interventions, whilst using demographic covariates to fine-tune local planning decisions rather than applying them directly across cycleways. This highlights that transferable modelling frameworks and the contextual calibration transferability strategy can support efficient cycling infrastructure planning, provided that demographic effects are treated as context-sensitive factors. Rather than ignoring demographic factors, these results highlight the need to use them carefully and locally, ensuring that planning decisions reflect cycleway-specific characteristics. Overall, this indicates that whilst cycleway intervention effects can be broadly transferred across contexts, the stability of demographic predictors seems more dependent on spatial similarity and local calibration.

Among transferability strategies, contextual calibration consistently outperforms direct transferability and local re-estimation. Its strength lies in selectively adapting parameters sensitive to local conditions whilst preserving structural insights from the source model. This avoids the rigidity of direct transferability and the inefficiency or overfitting risk of local re-estimation. Methodologically, contextual calibration may reflect principles of transfer learning and domain adaptation, where prior models are used but flexibly adjusted to new contexts. It is particularly relevant in cycleway intervention modelling, where marginal effects often depend on local features, such as built environment, infrastructure connectivity, or cycling culture. Practically, contextual calibration can reduce the need for extensive data and time requirements during model development, making it suitable for application in cycleways with limited monitoring capacity. It can also support consistency across comparative evaluations whilst remaining sensitive to local variation. As such, contextual calibration can achieve a crucial balance between transferability and specificity—an increasingly important consideration in data-driven urban active transport planning.

Transferability of intervention models also depends on the structure and assumptions of the modelling framework. Our comparative analysis of the ARIMAX, GAM, and GAMM+ARMA frameworks indicates that frameworks with greater structural flexibility achieve superior generalisation across spatial and temporal contexts. The relatively poor transferability of ARIMAX can be explained by its parametric nature and rigid temporal structure, whereby its orders (*p*,* d*,* q*) and coefficients are tightly coupled to the temporal dependence and noise characteristics of the source series^28^. When the target context differs substantially, such as due to shifts in cycling behaviour or environmental conditions, these parameters fail to capture local dynamics, resulting in minimal improvements from contextual calibration over local re‑estimation. This observation is consistent with prior studies that highlight the context‑dependence of classical time‑series models^29^ and explains why ARIMAX is mainly suitable for relatively stable environments.

In contrast, GAM showed higher transferability performance because of its flexible smooth terms capturing non-linear time trends^30^, which allow key intervention effects to be preserved whilst target‑specific temporal patterns are adapted. This flexibility leads to higher transferability performance observed for GAM in heterogeneous contexts. GAMM+ARMA further improves adaptability by modelling temporal autocorrelation and station‑specific random effects^10,25^, which effectively separates stable intervention effects from context‑dependent variation. Our findings are consistent with the transfer learning literature, which suggest that adaptation is most effective when domain‑invariant and domain‑specific components can be separated^21^.

Our findings also indicate that spatial transferability is more challenging than temporal transferability. Comparing spatial and temporal transferability does not imply equivalence between the two transferability types but rather using a consistent evaluation framework to examine how spatial versus temporal heterogeneity affects model performance under different planning contexts in cycling infrastructure evaluation. Temporal transferability tests stability under changing conditions at a fixed location, whereas spatial transferability tests generalisability across heterogeneous urban contexts whilst keeping the time period fixed. The comparison is, therefore, not intended to assess which form of transferability is superior, but rather to examine how alternative transferability strategies (direct transferability, contextual calibration, and local re-estimation) perform under different transferability contexts. To ensure comparability, model performance in spatial and temporal transferability contexts is evaluated using the same set of goodness-of-fit metrics and predictive accuracy. Thus, it allows a consistent assessment of model robustness and how intervention effects estimated in a source cycleway remain informative when applied across space or over time. By maintaining a common evaluation framework whilst recognising that spatial and temporal transferability address different sources of variation, the analysis allows transferability performance to be compared across contexts.

In the spatial transferability experiments across Cycleways 1 and 3, models exhibited higher RMSE and MAE values and greater variability in covariate effects, with some demographic factors, such as commuting distance and rental tenure, occasionally reversing direction. In contrast, temporal transferability within the same cycleway showed lower RMSE and more consistent parameter estimates, reflecting that the model structure remains broadly robust over time with minimal recalibration. These differences likely arise from spatial heterogeneity, affecting stability of model parameters and relationships between demographic features and bike-share usage. These variations not only affect the magnitude of cycleway intervention effects but also influence how demographic factors interact with the intervention variables, leading to instability in covariate direction and significance^27^. Prior study^20,31^ suggest that spatial differences can significantly reduce model accuracy when directly transferred without adjustment, necessitating strategies such as partial calibration or hierarchical modelling. Consequently, spatial transferability typically requires structural adjustments or local recalibration to avoid misrepresenting local cycling dynamics.

In contrast, temporal transferability tends to be more stable, especially when the spatial context remains unchanged. Key factors of cycling behaviour, such as docking stations accessibility, cycling infrastructure availability, and sociodemographic patterns, often change more gradually over time than across space. This temporal continuity has been observed in multiple studies. For instance, previous studies found that although model coefficients may shift over time, predictive performance remains adequate with limited updating, particularly when using calibration-based strategies^15,32^. Another study showed that temporal transferability in travel demand models can be achieved when local conditions change gradually over time^17^. In this context, contextual calibration provides a practical strategy to account for gradual temporal changes without re-estimating entire models. These differences suggest that strategy selection should be sensitive to the type of transferability task. For spatial transferability, more extensive recalibration or flexible model structures may be required to account for geographic heterogeneity. Conversely, temporal transferability can typically rely on minimal calibration that improves modelling efficiency and reduces implementation time. Recognising these differences can improve modelling framework design and strengthen the validity of cycleway intervention evaluations.

Building on these analytical findings, the study provides several practical implications for urban cycling infrastructure planning in urban areas. The positive effect of cycleway implementation across modelling frameworks and transferability contexts highlights the value of investing in dedicated cycling infrastructure, as interventions can increase bike‑share usage under varying spatial and temporal settings. Moreover, the strong performance of contextual calibration combined with flexible modelling frameworks highlights its potential as an adaptive and generalisable method for cycleway planning and evaluation. By reusing existing models with targeted local calibration, planners can generate early and reliable predictions of bike‑share responses to new or extended cycleways without the cost and delay of developing new models from scratch. In cycleways where the spatial context remains stable, even a short pre‑intervention or early post‑intervention window is sufficient to adapt the model, allowing policymakers to make timely decisions on maintenance, supporting cycling facilities, and design optimisations without waiting for long-term data. When cycleways are implemented in new geographic areas, contextual calibration allows the same framework to evaluate intervention effects across sites, supporting cycleway prioritisation, phased network expansion, and efficient resource allocation. By maintaining interpretability whilst reducing data and time requirements, this integrated transferability strategy ensures that modelling insights translate into operational and cost‑effective guidance for urban cycling network expansion.

From a methodological perspective, the modelling choices in this study are aligned with the main objective to evaluate the spatial and temporal transferability of cycleway intervention effects, rather than to optimise predictive performance. Classical time series and semi-parametric frameworks, including ARIMAX, GAM, and GAMM+ARMA, are well-established methodologies in Interrupted Time Series analysis. They are particularly appropriate for this study because they allow transparent estimation of intervention effects, support clear interpretation across different spatial and temporal contexts, and account for temporal dependence and the timing of policy interventions. These properties are essential when evaluating whether estimated intervention effects and key covariates maintain stability and interpretability when transferred across spatial and temporal contexts.

Nevertheless, extant research has significantly focussed on using machine learning advancements for this purpose. For instance, a study^33^ developed machine learning models to predict ride-hailing demand using random forest, extreme gradient boosting and neural network models, and found that a transfer learning method improved the prediction performance when applied across cities, suggesting the applicability of machine learning models in spatial transferability analyses. Similarly, another study^34^ evaluated the spatial transferability of pedestrian trip generation models and found that random forest exhibited stronger transferability across cities than some traditional statistical models, indicating the potential for machine learning approaches to capture complex spatial patterns across contexts. Building on these studies, future research could extend our work by incorporating more granular cycling behavioural data and by exploring machine learning models (e.g., random forest, extreme gradient boosting, neural networks)^33,34^. Further, a new modelling approach (hybrid of classical and machine learning) can also be introduced, whereby data-driven models can capture complex, non-linear demand patterns whilst classical time series models provide interpretable estimates of intervention effects, and their combined usage strengthens the evaluation of model transferability for cycling infrastructure planning.

In summary, this study evaluated the spatial and temporal transferability of cycleway intervention models using ARIMAX, GAM, and GAMM+ARMA combined with three transferability strategies. Contextual calibration consistently achieved the best balance between transferability and interpretability, whilst intervention effects were broadly transferable but demographic features were more sensitive to spatial heterogeneity. These findings highlight the value of adaptive modelling strategies for efficient and generalisable cycling infrastructure planning. Future research could extend this work by incorporating microscopic data to better capture behavioural heterogeneity, and by exploring machine learning approaches that may uncover complex, non-linear relationships beyond the traditional statistical frameworks. Such developments would further enhance the adaptability and robustness of modelling tools for active transport planning.

## Methods

### Data and pre-processing

#### Spatial transferability

To examine the transferability of cycleway intervention models across spatial contexts, this transferability analysis incorporates three cycleways: Cycleway 1 (C1), Cycleway 3 (C3), and Cycleway 6 original segment (C6). All three cycleways were implemented around the same time in 2016, providing a suitable experimental framework for spatial comparison. Cycleway 1 (from White Hart Lane in Tottenham to Liverpool Street station) and Cycleway 3 (from Tower Gateway to Parliament Square) were implemented in April 2016 and are used as target cycleways, representing distinct spatial contexts. Cycleway 6 original segment, connecting Elephant and Castle to Stonecutter Street, was implemented in May 2016, and serves as the source cycleway. Whilst London has developed approximately 30 different cycleways since 2012, only a limited subset is suitable for robust intervention modelling and transferability analysis. Many cycleways (i) lack publicly documented implementation dates (key for intervention analysis), (ii) were delivered through multiple overlapping phases, and (iii) experienced gradual infrastructure changes that prevent identification of a clear intervention point. For the study period, Cycleways 1, 3 and 6 were selected because they provided accurate and publicly documented implementation dates and consistent monitoring data on before and after implementation, making them appropriate for developing intervention models and analysing spatial and temporal transferability. Further, Cycleway 1 and 3 were selected as target cycleways because they differ systematically in spatial configuration, land-use context, and cycling demand. Cycleway 1 is a north-south commuter corridor serving dense residential and mixed-use neighbourhoods, whereas Cycleway 3 is an east-west corridor connecting major employment centres along the Thames River. Cycleway 6 was used as the source cycleway because it provides a relatively stable post-intervention period, making it well-suited for parameter estimation before spatial transferability.

Despite these differences, Cycleways 1, 3, and 6 show several key similarities that support meaningful comparison. All three cycleways are well-established and widely used in London and form part of Transport for London’s strategic cycleway network. They have high-quality segregated infrastructure, serve high-demand cycling corridors, and are located in dense inner-London environments, with strong integration into major transport hubs (e.g., King’s Cross, Elephant & Castle, Tower Gateway). They also have sufficient observed bike-share trip volumes before and after cycleway implementation, ensuring that differences in model performance are not driven by data quality or scale effects, but instead reflect contextual variation related to transferability evaluation. Overall, the selection of Cycleways 1, 3, and 6 is based on empirical and data-related considerations and does not constrain the interpretation of transferability results. Instead, this selection allows the transferability analysis to be informed by comparable data conditions whilst capturing contextual differences across cycleways.

**Fig. 1:**
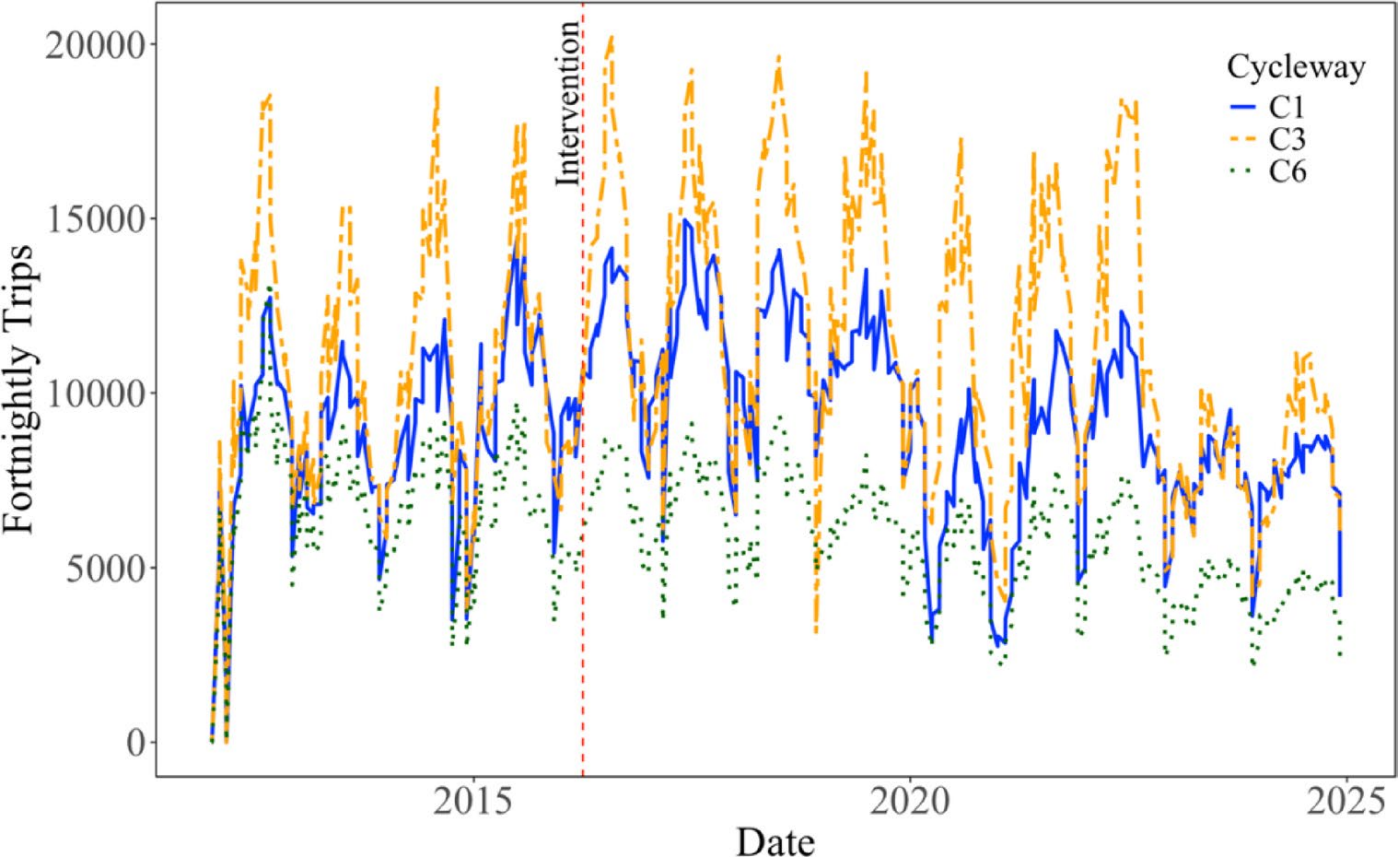
Fortnightly bike-share trips over time (Cycleway 1, Cycleway 3, Cycleway 6) for spatial transferability.

Bike-share trip data were sourced from Transport for London, which provides publicly accessible records from the Santander Cycles system. Trip data were extracted from docking stations located within 300 m of each cycleway—a buffer distance consistent with earlier work^25^, which suggests a 300 m radius as a typical influence zone of cycleway corridors. For the spatial transferability analysis, data were obtained from 16, 15, and 15 docking stations for C1, C3 and C6. Trip counts were aggregated fortnightly per station, resulting in a panel dataset with consistent temporal granularity. The dataset for each cycleway spans from 2012 to 2024, capturing multi-year trends in bike-share usage before and after cycleway implementation. Intervention dates and observation periods were aligned across all transferability strategies to ensure consistency in design and support meaningful comparison. Figure 1 presents fortnightly bike-share trips across three cycleways, with the intervention date marked for each. These time series charts clarify the temporal patterns in the dependent variable before and after the intervention.

#### Temporal transferability

**Fig. 2:**
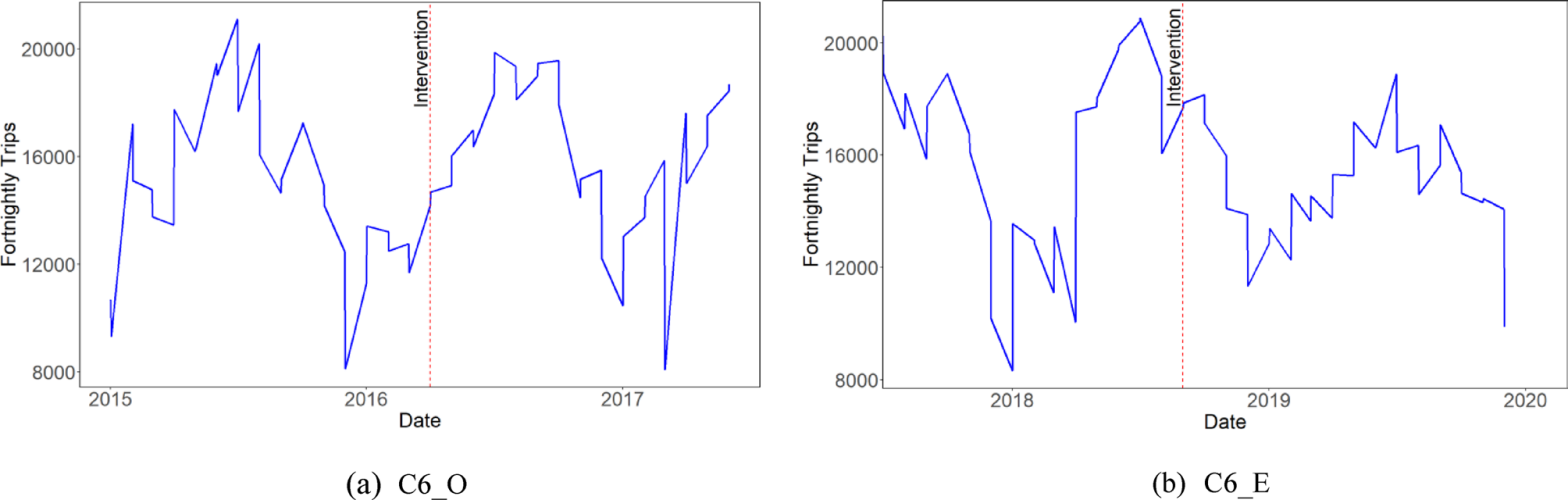
Fortnightly bike-share trips over time (Cycleway 6 original [C6_O] and Cycleway 6 extension [C6_E] for temporal transferability.

To investigate the stability and transferability of cycleway implementation effects over time, a temporal transferability design was developed using two phases of Cycleway 6. The original segment (Elephant and Castle to Stonecutter Street) was opened in May 2016, whilst an extension (between Farringdon and King’s Cross) opened in September 2018. In this analysis, the earlier segment serves as the source model, and the transferability is tested on the later extension. Fortnightly trip data were obtained from 32 docking stations located within 300 m of the full Cycleway 6 corridor, covering the original (C6_O) and extended segments (C6_E). To ensure a strict temporal separation, the source dataset spans from January 2015 to June 2017, and the target dataset covers July 2017 to December 2019. The source period ends before September 2018 (when the extension segment opened) to avoid contamination from post-intervention exposure. As the source data were collected before the COVID-19 pandemic, the target period was also limited to December 2019 to ensure that observed effects are not influenced by pandemic-related disruptions. The temporal transferability assesses whether bike-share usage patterns derived from an earlier intervention can be generalised to a later phase of the same cycleway under similar spatial conditions and cycling demand. Figure 2 presents fortnightly bike-share trips across full Cycleway 6, with the intervention date marked for each.

#### Demographic features

**Fig. 3:**
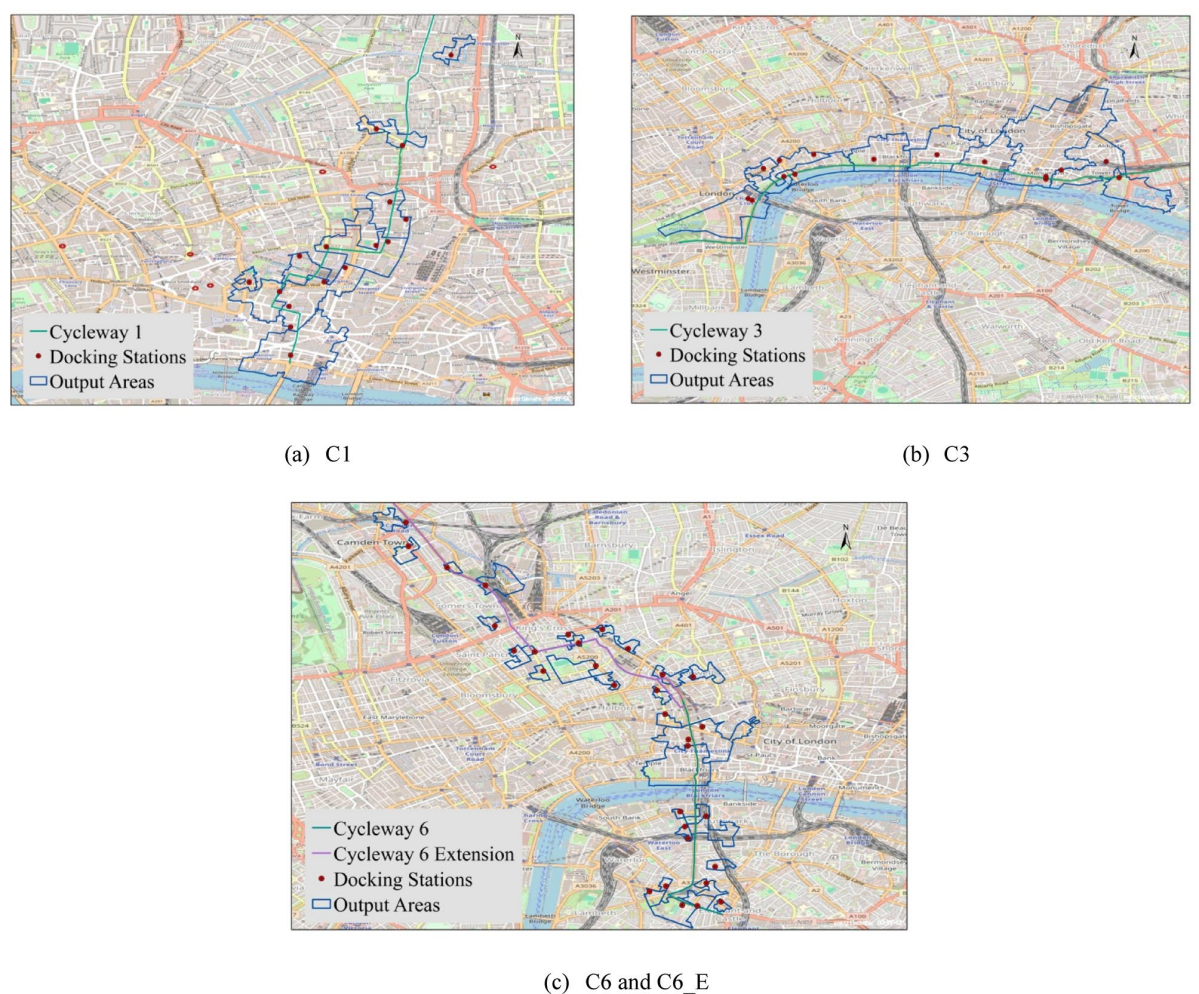
Maps of Cycleways (C6, C6 extension, C1, C3), docking stations (shown in red dots), and the corresponding Output Areas (shown in blue boundary areas). The map was created by the authors using ArcMap 10.8.0.12790 in ArcGIS Desktop 10.8 (Esri, Redlands, CA, USA; https://www.esri.com/), with OpenStreetMap as the basemap. Basemap data © OpenStreetMap contributors, available under the Open Database License (https://www.openstreetmap.org/copyright).

Demographic variables were obtained from the 2021 UK Census at the Output Area level and spatially matched to each docking station, with each station assigned the characteristics of the Output Area where it is located. Whilst the bike-share dataset spans 2012–2024, the 2021 Census provides the most recent and detailed demographic information available and reflects the contextual characteristics of the area surrounding each station. To improve model robustness and minimise collinearity issues, a two-step variable selection approach was employed. In the first stage, covariates with variance inflation factor values exceeding 5 were excluded from further analysis. In the second stage, a stepwise regression method based on the Akaike Information Criterion was used to select the most informative subset of variables. Following this process, six key demographic features were selected: (i) age and gender, (ii) commuting distance (proportions travelling less than 2 km and between 2 and 5 km), (iii) travel mode to work (proportion of bicycle commuters), (iv) housing tenure (owned and rented accommodations), and (v) economic activity status (proportions employed and unemployed, excluding full-time students). Including these demographic attributes helps explain variations in bike-share usage across different areas and allows the model to account for underlying social and economic conditions. In particular, the method to travel to work variable is used as an area-level demographic indicator of the pre-existing modal share of commuting around each cycleway, reflecting longer-term differences in local travel behaviour rather than individual commuting choices. It serves as a contextual control for spatial differences across cycleways, rather than a time-varying behavioural variable. Under this interpretation, the use of the 2021 Census in the main analysis is appropriate, as it provides the most recent and comprehensive demographic information at the required spatial resolution. Descriptive statistics for these variables are summarised in Table 6. The spatial layout of cycleways, selected docking stations, and corresponding Output Areas are illustrated in Fig. 3. Whilst the use of aggregated data limits the ability to capture individual-level behaviours and introduces the risk of ecological fallacy, it remains a valuable approach for informing transport policy, which is typically developed at aggregate spatial levels such as communities, counties, or cities^10^.

**Table 6:** Descriptive statistics of the model dependent and independent variables.

Variable	Description	Cycleway 1		Cycleway 3		Cycleway 6		Cycleway 6 Extension	
		Mean (S.D.)	Min (Max)	Mean (S.D.)	Min (Max)	Mean (S.D.)	Min (Max)	Mean (S.D.)	Min (Max)
Bike-share trips	Fortnightly trips	574 (382.5)	0 (2108)	741 (433.4)	0 (2913)	402.5 (283.6)	0 (3294)	486.4 (239.8)	0 (1484)
Method to travel to work	The percentage of bicycle commuters	3.1 (1.9)	0.9 (8.5)	2.5 (1.2)	0.8 (5.1)	5.2 (3.1)	1.6 (10.7)	5.1 (2.7)	1.2 (10.7)
Distance to work less than 2 km	The percentage of individuals commuting less than 2 km	11.4 (3.1)	7.1 (16.1)	14.1 (2.6)	9.0 (18.7)	11.1 (4.1)	3.5 (16.4)	13.6 (5.6)	3.5 (27.7)
Distance to work between 2–5 km	The percentage of individuals commuting between 2–5 km	7.0 (4.7)	3.4 (18.1)	7.9 (3.2)	5.0 (16.4)	10.8 (5.5)	3.6 (19.2)	11.6 (4.9)	3.6 (21.8)
Female	The percentage of females	45.2 (4.1)	38.8 (52.1)	46.1 (8.4)	35.1 (64.3)	49.1 (4.9)	40.9 (55.4)	50.3 (4.1)	40.9 (58.2)
Age between 20–49	The proportion of individuals between 20 to 49	61.3 (11.9)	39.6 (83.1)	66.8 (11.5)	11.6 (86.4)	54.6 (11.4)	40.4 (81.6)	54.1 (10.6)	36.3 (81.6)
Age between 50–74	The proportion of individuals between 50 to 74	21.3 (9.5)	8.5 (42.8)	20.6 (6.9)	35.1 (39.4)	26.1 (9.3)	5.0 (35.8)	23.2 (9.3)	2.3 (39.8)
Employed	The proportion of individuals employed	65.6 (13.7)	33.2 (81.3)	61.2 (15.4)	37.9 (83.1)	55.8 (14.2)	15.5 (74.9)	50.7 (16.3)	7.3 (74.9)
Unemployed	The proportion of individuals unemployed	2.8 (1.6)	0.7 (6.6)	2.1 (1.1)	0 (3.8)	2.5 (1.8)	1.0 (7.5)	3.8 (2.36)	0.5 (7.9)
Owned	The proportion of owned households	34.5 (13.0)	12.2 (63.6)	29.2 (14.5)	14.4 (57.6)	31.5 (15.4)	3.9 (50.5)	25.9 (13.9)	3.9 (50.5)
Rented	The proportion of rented households	65.3 (12.9)	36.4 (87.8)	70.6 (14.6)	42.3 (85.6)	68.4 (15.4)	49.5 (96.1)	73.6 (13.5)	49.5 (96.1)

### Modelling frameworks

This study evaluates the spatial and temporal transferability of models developed to estimate the effects of cycleway implementation on bike-share usage in London. The analysis focusses on three modelling frameworks described below, which differ in structural flexibility in capturing temporal autocorrelation, non-linear trends, and spatial heterogeneity. Model performance is assessed under different transferability strategies to examine how well models developed in one spatial or temporal context generalise to others.

All models are estimated using an interrupted time series design to evaluate the immediate and sustained impacts of cycleway implementation on bike-share usage^35,36^. Two intervention terms are included: $\:{D}_{t}$: a binary indicator set to 1 after the intervention and 0 otherwise, capturing the immediate change. $\:{P}_{t}$: a continuous variable counting the time since the intervention (0 before), reflecting the sustained post-intervention effect. $\:{Y}_{it}$ denotes the observed number of bike-share trips from station $\:i$ at time $\:t$. Each model includes a set of covariates ($\:{\mathbf{{\rm\:X}}}_{it}$) incorporating demographic features relevant to bike-share usage.

The Autoregressive Integrated Moving Average model with exogenous variables (ARIMAX) is used. To stabilise variance and satisfy model assumptions, the dependent variable is log-transformed, and model parameters are estimated using maximum likelihood. ARIMAX is well-suited to time series exhibiting stable temporal patterns and autocorrelated residuals. The model is formulated as

1 $$\:log\left({Y}_{it}+1\right)={\beta\:}_{0}+{{\beta\:}_{1}{T}_{t}+\beta\:}_{2}{D}_{t}+{\beta\:}_{3}{P}_{t}+{\gamma\:}^{T}{\boldsymbol{{\rm\:X}}}_{it}+{\eta\:}_{t},$$

2 $$\:{\eta\:}_{t}=\sum\:_{j=1}^{p}{\varnothing\:}_{j}{\eta\:}_{t-j}+\sum\:_{k=1}^{q}{\theta\:}_{k}{\epsilon\:}_{t-k}+{\epsilon\:}_{t},$$

where, $\:{T}_{t}$ is a continuous variable denoting a linear time trend since the start of the observation period. Intervention effects are quantified by $\:{\boldsymbol{\beta\:}}_{2}$ (immediate change) and $\:{\boldsymbol{\beta\:}}_{3}$ (post-intervention slope). The error term $\:{\eta\:}_{t}$ follows an ARMA (*p*,* q*) process to account for autocorrelation, where $\:{\epsilon\:}_{t}$ is white noise. The ARMA error structure is introduced to capture serial correlation in bike-share usage that cannot be fully explained by observed covariates and cycleway intervention terms. In the context of interrupted time series analysis, ignoring residual autocorrelation may lead to biased standard errors and potentially overestimated intervention effects.

The ARIMAX model’s rigid assumption of a linear temporal trend may reduce its flexibility in contexts with heterogeneous residual structures, thereby potentially affecting its transferability. To this end, a Generalised Additive Model (GAM) addresses the ARIMAX model’s limitation in capturing non-linear time trends in bike-share usage. Cycling activity is often influenced by seasonality, behavioural shifts, and irregular fluctuations that may not follow a linear trend. Although such complexity can be approximated in ARIMAX models using polynomial terms, these adjustments require prior specifications and may poorly represent unknown patterns. GAM extends generalised linear models by introducing smooth spline functions in place of fixed time trends^37^, allowing temporal structure to be learned directly from the data with minimal parametric assumptions. In this study, the GAM is estimated using a Poisson distribution, which is appropriate for modelling count outcomes. Intervention terms and covariates are retained in the additive predictor alongside a smooth function of time trend. The model is specified as

3 $$\:{Y}_{it}\:\sim\:Poisson\:\left({\lambda\:}_{it}\right),$$

4 $$\:log\left({\lambda\:}_{it}\right)={\beta\:}_{0}+s\left({T}_{t}\right)+{\beta\:}_{1}{D}_{t}+{\beta\:}_{2}{P}_{t}+{\gamma\:}^{T}{\boldsymbol{{\rm\:X}}}_{it},$$

where, $\:{\:\lambda\:}_{it}$is the expected values of bike-share trips, $\:s\left({T}_{t}\right)$ denotes a smooth function of the continuous time trend variable $\:{T}_{t}$, capturing complex non-linear trends and seasonality. All other variables are defined previously.

A limitation of GAM is that it assumes a common temporal trend across all stations, which may reduce its spatial generalisability in transferability settings. The Generalised Additive Mixed Model with autoregressive and moving average (GAMM+ARMA) extends GAM by allowing for station-specific temporal trends and autocorrelated errors to capture spatial heterogeneity in bike-share usage affected by differences in cycling demand, land use, or accessibility. The GAMM+ARMA introduces random smooth functions ($\:{s}_{i}\left({T}_{t}\right)$) to capture station-level variation and incorporates an ARMA error structure to model temporal dependence that remains after accounting for smooth non-linear trends and station-specific effects. This specification allows the model to distinguish between temporal patterns captured by smooth functions and short-term serial correlation in unexplained residuals, which is particularly important when transferring models across heterogeneous spatial or temporal contexts. By combining non-linear time trends, spatial heterogeneity, and autocorrelated residuals, GAMM+ARMA provides a flexible framework for modelling complex spatiotemporal patterns and evaluating transferability in diverse contexts. The model is specified as

5 $$\:log\left({\lambda\:}_{it}\right)={\beta\:}_{0}+{s}_{i}\left({T}_{t}\right)+{\beta\:}_{1}{D}_{t}+{\beta\:}_{2}{P}_{t}+{\gamma\:}^{T}{\mathbf{{\rm\:X}}}_{it}+{\eta\:}_{it},$$

6 $$\:{\eta\:}_{it}=\sum\:_{j=1}^{p}{\varnothing\:}_{i}{\eta\:}_{i,t-i}+\sum\:_{k=1}^{q}{\theta\:}_{j}{\epsilon\:}_{i,t-j}+{\epsilon\:}_{it}.$$

### Transferability contexts

To evaluate transferability of cycleway intervention models, this study considers two transferability contexts: spatial and temporal. Building on the general definition of transferability as the extent to which a model developed in one context remains valid in another similar context, in this study, transferability refers to whether relationships and intervention effects estimated for one cycleway remain informative when applied to other cycleways or to a later time period. Following the concept of model transference discussed by Ortúzar and Willumsen^38^, transferability should not be interpreted as the strict stability or equality of parameter values across contexts. Instead, transferred parameters are expected to be context-dependent and to serve as informative inputs that may require calibration when applied elsewhere. This perspective is particularly relevant for cycleway interventions, where cycling demand, built environment, and demographic characteristics can vary across locations or change over successive implementation stages. Accordingly, transferability in this study is framed as a practical strategy for extending useful information from a data-rich cycleway to other cycleways or to later phases of the same cycleway. In this framework, spatial and temporal transferability are treated as two distinct but complementary transferability contexts for evaluating model robustness and the performance of transferability strategies.

In the transport modelling literature, spatial transferability generally refers to the extent to which models or behavioural relationships estimated in one spatial context can be applied to other spatial contexts with different local characteristics, whilst retaining acceptable explanatory or predictive validity^39^. In this study, spatial transferability focusses on whether models estimated for one cycleway can provide useful information when applied to other cycleways located in different spatial contexts. This transferability is important for cycling infrastructure evaluation because new cycleways are often implemented gradually across a city, whilst detailed post-intervention data are typically available for only a small number of cycleways. Spatial transferability allows intervention effects estimated from a source cycleway to be applied to other cycleways where local data are insufficient for independent model estimation. In addition, spatial transferability tests whether cycling behavioural relationships, such as the magnitude and temporal pattern of bike-share ridership following cycling infrastructure implementation, remain informative across cycleways with different local demographic contexts. In the spatial transferability context, models are trained on the Cycleway 6 original segment (C6) and applied to Cycleway 1 (C1) and Cycleway 3 (C3).

Temporal transferability is commonly understood as the extent to which behavioural relationships or model parameters estimated at one point in time remain applicable or stable when used to represent behaviour in subsequent time periods, despite potential temporal evolution in preferences or responses^40^. In this study, temporal transferability focusses on whether models estimated at an earlier stage of a cycleway intervention remain informative when applied to a later stage (or time) of the same cycleway. This transferability is particularly important for cycling infrastructure evaluation because cycleway investments are typically implemented incrementally, and decisions regarding extensions are often made before long-term post-intervention effects have been fully stabilised. Consequently, information from early implementation phases is frequently used to inform subsequent planning decisions, even though cycling behaviour may continue to evolve over time. In this study, temporal transferability evaluates whether models estimated on the original segment of Cycleway 6 perform consistently when applied to its later extension. By holding the spatial context constant, this transferability context focusses on how cycling responses change over time, and on whether early stage intervention effects remain relevant as the cycleway is extended. In the temporal transferability context, models estimated on the original segment of Cycleway 6 are applied to a later extension of the same cycleway. To support a consistent evaluation across transferability contexts, each modelling approach is assessed under spatial and temporal transferability using the same set of performance metrics.

### Transferability strategies

**Fig. 4:**
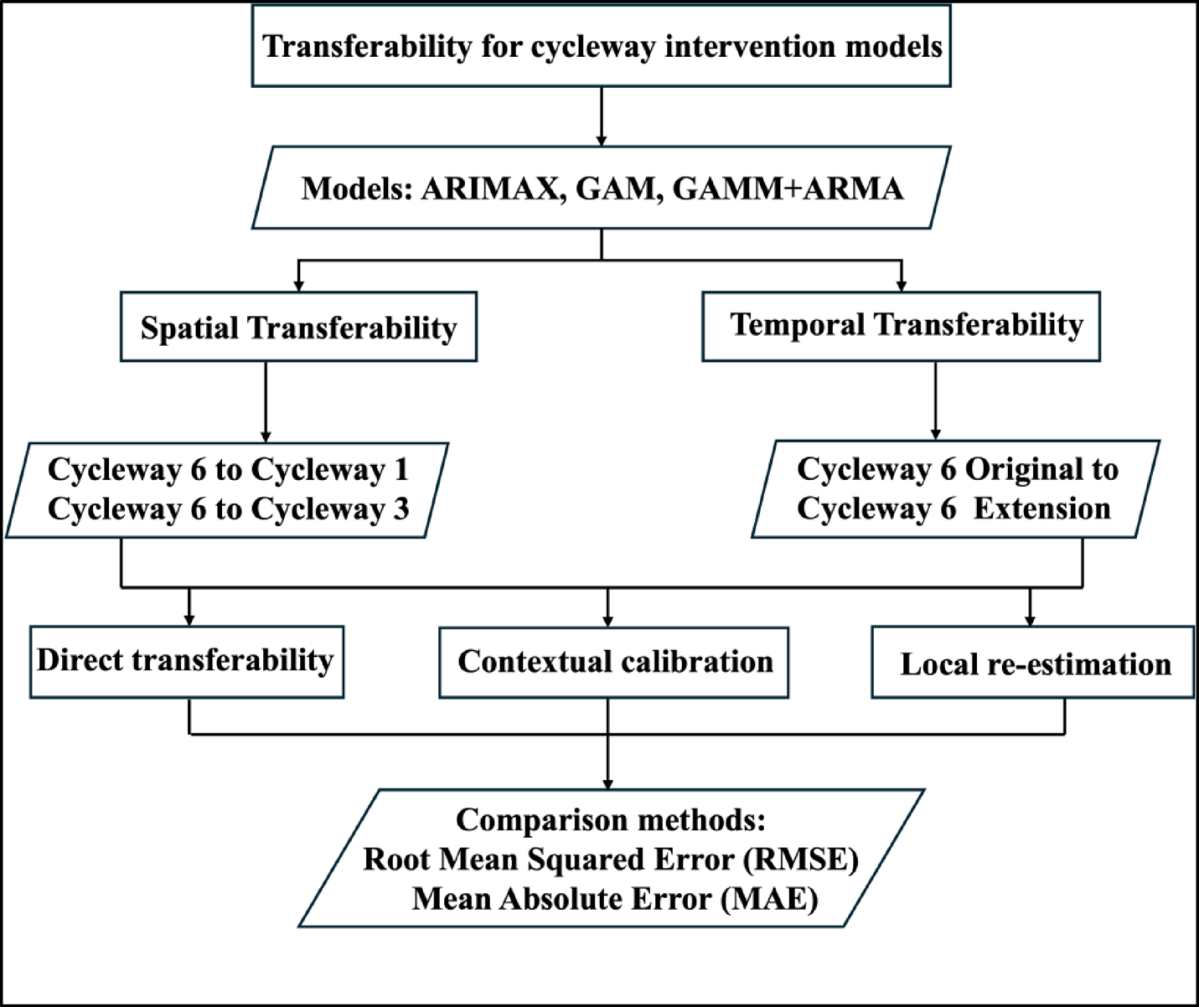
Transferability evaluation framework for cycleway intervention models.

To evaluate model transferability across spatial and temporal contexts, we considered three strategies: direct transferability, contextual calibration, and local re-estimation. These strategies were used across all modelling frameworks and reflect different levels of adaptation. They also represent varying degrees of reliance on prior model knowledge from the source context and provide a structured basis for evaluating model robustness and generalisability in cycling infrastructure intervention settings. Figure 4 illustrates the framework for evaluating cycleway intervention model transferability.

Direct transferability is the most straightforward approach, implying that the full model specification and estimated parameters from the source context are applied directly to the target context without any modification^39^. For the ARIMAX model, this process involves using the same autoregressive and moving average terms, and covariate coefficients estimated from the source cycleway. For the GAM and GAMM models, all smooth terms and fixed effects are retained as fitted in the source model. This strategy tests the model’s robustness under zero-adaptation and provides a baseline for assessing the value of contextual adjustments. Whilst this approach is efficient by reusing the source model, its performance may be limited when the source and target contexts differ significantly.

The contextual calibration strategy allows for partial adaptation by retaining the structure from the source model whilst calibrating context-sensitive parameters. In the ARIMAX framework, this process fixes some covariates and re-estimates intervention-related and temporal coefficients based on the target data. A custom ARIMAX fitting function is used to calibrate model parameters. In the GAM, fixed-effect coefficients from the source model are used to initialise estimation in the target data, whilst smooth terms are re-estimated using restricted maximum likelihood to improve model stability. For the GAMM+ARMA model, contextual calibration retains autoregressive structure and random effects specification, and calibrates fixed effects and smooth terms. Source-estimated fixed-effect coefficients are used as initial values, and the model is estimated using restricted maximum likelihood with additional convergence controls set on the expected maximisation algorithm to ensure model stability. Contextual calibration provides a balanced approach that combines prior model knowledge with targeted adjustment. It improves contextual applicability and reduces the need to fully re-estimate the model parameters, making it efficient for model development under tight implementation timelines.

Unlike previous strategies, local re-estimation does not rely on any source-derived information. Instead, it re-estimates the model from scratch using the target dataset. Whilst the source model specification is retained, all parameters are re-estimated independently to reflect the specific characteristics of the target data. Candidate covariates are selected based on those used in the source model. Multicollinearity is then carefully assessed to ensure estimation stability within the local context. This approach maintains consistency with the original model framework and allows improved model interpretability in the local context. Further, this strategy offers maximum flexibility to capture local variation, but it also needs complete re-fitting, which may be limited in data availability or computational resources.

All analyses were conducted in *R*-studio (version 4.4.1), using the packages *forecast*, *mgcv*, *nlme*, *MASS*, and *lmtest* for modelling, *dplyr*, *tidyr*, *stringr*, *lubridate*, and *readr* for data processing. ArcGIS 10.8 and ggplot2 were used for visualisation. To facilitate reproducibility, all codes and associated data are provided via GitHub.

## Data Availability

The raw data used in this study are available from Transport for London’s open data portal (https://cycling.data.tfl.gov.uk). The processed datasets generated and analysed during the current study are available from Dr. Haitao He upon reasonable request.

## Appendix

See Tables 7, 8, 9, 10, 11, 12, 13, 14, 15, 16, 17, 18, 19, 20, 21.

**Table 7:** Results of the ARIMAX models under different transferability strategies for spatial transferability.

Transferability strategies	Cycleway 1			Cycleway 3		
	DT	CC	LRE	DT	CC	LRE
	Estimate (S.E.)	Estimate (S.E.)	Estimate (S.E.)	Estimate (S.E.)	Estimate (S.E.)	Estimate (S.E.)
Intercept	0.5599 (0.5808)	–	2.7674 (0.8143)	0.5599 (0.5808)	–	3.3249 (0.5924)
Post-intervention indicator	–	–	–	–	–	–
Time-since-intervention	− 0.0208 (0.0014)	− 0.0245 (0.0016)	− 0.0443 (0.0064)	− 0.0208 (0.0014)	− 0.0174 (0.0005)	− 0.0447 (0.0053)
Method to travel to work (bicycle)	− 0.0467 (0.0088)	− 0.1069 (0.0150)	− 0.2588 (0.0536)	− 0.0467 (0.0088)	–	
Distance to work less than 2 km	− 0.0050 (0.0024)	0.0147 (0.0022)	–	− 0.0050 (0.0024)	− 0.0167 (0.0020)	− 0.0276 (0.0068)
Distance to work between 2–5 km	− 0.0415 (0.0026)	–	–	− 0.0415 (0.0026)	–	–
Female	0.0852 (0.0057)	–	–	0.0852 (0.0057)	–	–
Age between 20–49	0.0234 (0.0043)	–	–	0.0234 (0.0043)	–	–
Age between 50–74	−	–	–	–	–	–
Employed	0.0090 (0.0023)	–	–	0.0090 (0.0023)	0.0260 (0.0016)	0.0171 (0.0073)
Rented	–	− 0.0226 (0.0013)	–	–	− 0.0134 (0.0015)	− 0.0123 (0.0074)
Model statistics						
Log-likelihood	–	− 8950.59	− 4663.24	–	− 7709.47	− 5132.91
Akaike information criterion	–	17915.19	9348.47	–	15432.94	10285.83
Pseudo R-squared	–	0.04	0.08	–	0.15	0.16
Number of observations	4976	4976	4976	4680	4680	4680

“-” not statistically significant or not included.

**Table 8:** Results of the GAM model under different transferability strategies for spatial transferability.

Transferability strategies	Cycleway 1			Cycleway 3		
	DT	CC	LRE	DT	CC	LRE
	Estimate (S.E.)	Estimate (S.E.)	Estimate (S.E.)	Estimate (S.E.)	Estimate (S.E.)	Estimate (S.E.)
Intercept	− 0.3145 (0.0845)	5.2096 (0.0665)	3.0046 (0.0655)	− 0.3145 (0.0845)	4.2710 (0.0643)	1.5376 (0.0622)
Post-intervention indicator	0.2812 (0.0065)	0.2260 (0.0051)	0.2249 (0.0051)	0.2812 (0.0065)	0.6595 (0.0049)	0.6564 (0.0049)
Time-since-intervention	0.0480 (0.0011)	0.0512 (0.0009)	0.0509 (0.0009)	0.0480 (0.0011)	0.0644 (0.0008)	0.0636 (0.0008)
Method to travel to work (bicycle)	− 0.0234 (0.0004)	− 0.0749 (0.0005)	− 0.0619 (0.0004)	− 0.0234 (0.0004)	0.0129 (0.0005)	–
Distance to work less than 2 km	0.0110 (0.0001)	− 0.0018 (0.0001)	0.0008 (0.0001)	0.0110 (0.0001)	− 0.0027 (0.0001)	0.0007 (0.0001)
Distance to work between 2–5 km	− 0.0333 (0.0001)	− 0.0021 (0.0001)	− 0.0050 (0.0001)	− 0.0333 (0.0001)	0.0381 (0.0001)	0.0213 (0.0001)
Female	0.0536 (0.0002)	− 0.0172 (0.0001)	–	0.0536 (0.0002)	− 0.0014 (0.0001)	–
Age between 20–49	0.0070 (0.0001)	− 0.0247 (0.0001)	–	0.0070 (0.0001)	− 0.0379 (0.0001)	–
Age between 50–74	0.0006 (0.0002)	− 0.0291 (0.0001)	–	0.0006 (0.0002)	− 0.0585 (0.0002)	–
Employed	0.0054 (0.0001)	0.0174 (0.0001)	0.0024 (0.0001)	0.0054 (0.0001)	0.0320 (0.0001)	0.0167 (0.0001)
Rented	− 0.0008 (0.0001)	− 0.0071 (0.0001)	− 0.0033 (0.0001)	− 0.0008 (0.0001)	− 0.0183 (0.0001)	− 0.0176 (0.0001)
Model statistics						
Log-likelihood	–	− 544418.5	− 565270.8	–	− 432949.7	− 476137.8
Akaike information criterion	–	1,088,877	1,130,576	–	865939.4	952307.6
Adjusted *R*-squared	–	0.165	0.141	–	0.323	0.269
Number of observations	4976	4976	4976	4680	4680	4680

**Table 9:** Results of the GAMM+ARMA model under different transferability strategies for spatial transferability.

Transferability strategies	Cycleway 1			Cycleway 3		
	DT	CC	LRE	DT	CC	LRE
	Estimate (S.E.)	Estimate (S.E.)	Estimate (S.E.)	Estimate (S.E.)	Estimate (S.E.)	Estimate (S.E.)
Intercept	− 0.6290 (0.1629)	5.2876 (0.1406)	3.1793 (0.1321)	− 0.6290 (0.1629)	5.4204 (0.1227)	2.5686 (0.1104)
Post-intervention indicator	0.1616 (0.0095)	0.0747 (0.0073)	0.0782 (0.0072)	0.1616 (0.0095)	0.4474 (0.0072)	0.4823 (0.0071)
Time-since-intervention	0.0534 (0.0022)	0.0499 (0.0019)	0.0498 (0.0019)	0.0534 (0.0022)	0.0523 (0.0016)	0.0538 (0.0015)
Method to travel to work (bicycle)	− 0.0217 (0.0008)	− 0.0745 (0.0013)	− 0.0606 (-0.0001)	− 0.0217 (0.0008)	0.0228 (0.0011)	–
Distance to work less than 2 km	0.0115 (0.0002)	− 0.0022 (0.0001)	–	0.0115 (0.0002)	− 0.0091 (0.0001)	− 0.0048 (0.0001)
Distance to work between 2–5 km	− 0.0317 (0.0002)	− 0.0025 (0.0003)	− 0.0061 (0.0003)	− 0.0317 (0.0002)	0.0309 (0.0002)	0.0145 (0.0002)
Female	0.0521 (0.0004)	− 0.0162 (0.0004)	–	0.0521 (0.0004)	− 0.0020 (0.0002)	–
Age between 20–49	0.0075 (0.0003)	− 0.0233 (0.0003)	–	0.0075 (0.0003)	− 0.0365 (0.0002)	–
Age between 50–74	0.0015 (0.0004)	− 0.0278 (0.0004)	–	0.0015 (0.0004)	− 0.0576 (0.0004)	–
Employed	0.0049 (0.0002)	0.0167 (0.0002)	0.0027 (0.0001)	0.0049 (0.0002)	0.0317 (0.0001)	0.0166 (0.0001)
Rented	− 0.0006 (0.0001)	− 0.0070 (0.0003)	− 0.0031 (0.0001)	− 0.0006 (0.0001)	− 0.0181 (0.0001)	− 0.0174 (0.0001)
Model statistics						
Log-likelihood	–	− 178384.5	− 201981.8	–	− 193966.3	− 201981.8
Akaike Information Criterion	–	356,799	403985.7	–	387962.6	403985.7
Adjusted *R*-squared	–	0.161	0.138	–	0.312	0.262
Number of observations	4976	4976	4976	4680	4680	4680

**Table 10:** Results of the three modelling frameworks under different transferability strategies for temporal transferability.

Transferability strategies	ARIMAX			GAM			GAMM+ARMA		
	DT	CC	LRE	DT	CC	LRE	DT	CC	LRE
	Estimate	Estimate	Estimate	Estimate	Estimate	Estimate	Estimate	Estimate	Estimate
Intercept	5.8350 (0.4878)	–	6.1054 (0.2364)	5.6876 (0.0397)	3.9318 (0.0776)	3.8263 (0.0774)	5.7007 (0.0651)	3.8895 (0.0938)	3.8876 (0.0849)
Post-intervention indicator	0.5071 (0.1091)	–	–	0.0791 (0.0106)	0.3163 (0.0106)	0.3163 (0.0106)	0.0957 (0.0093)	0.3139 (0.0095)	0.3157 (0.0089)
Time-since-intervention	− 0.0119 (0.0070)	− 0.0079 (0.0021)	–	0.0896 (0.00740	0.2314 (0.0086)	0.2314 (0.0086)	0.0880 (0.0072)	0.2391 (0.0082)	0.2333 (0.0073)
Method to travel to work (bicycle)	0.2056 (0.0168)	–	–	0.0330 (0.0006)	0.0115 (0.0006)	–	0.0297 (0.0020)	− 0.0068 (0.0022)	–
Distance to work less than 2 km	0.0213 (0.0053)	0.0150 (0.0013)	0.0131 (0.0026)	0.0175 (0.0001)	0.0171 (0.0001)	0.0176 (0.0001)	0.0162 (0.0006)	0.0156 (0.0006)	0.0155 (0.0006)
Distance to work between 2–5 km	− 0.0455 (0.0066)	− 0.0080 (0.0019)	− 0.0101 (0.0034)	− 0.0156 (0.0002)	− 0.0048 (0.0002)	− 0.0050 (0.0002)	− 0.0151 (0.0007)	− 0.0044 (0.0008)	− 0.0044 (0.0008)
Rented	− 0.0089 (0.0026)	− 0.0013 (0.0008)	− 0.0030 (0.0012)	− 0.0042 (0.0001)	− 0.0039 (0.0001)	− 0.0033 (0.0001)	− 0.0037 (0.0002)	− 0.0039 (0.0003)	− 0.0035 (0.0002)
Model statistics									
Log-likelihood	–	− 1925.06	− 1911.43	–	− 85373.24	− 85547.29	–	− 14414.3	− 14913.7
Akaike Information Criterion	–	3864.11	3840.86	–	170778.5	171124.6	–	28852.6	29847.41
Adjusted *R*-squared	–	0.08	0.09	–	0.331	0.33	–	0.328	0.326
Number of observations	1888	1888	1888	1888	1888	1888	1888	1888	1888

**Table 11:** RMSE and MAE of three modelling frameworks across transferability strategies and contexts.

Cycleway #	ARIMAX			GAM			GAMM+ARMA		
	DT	CC	LRE	DT	CC	LRE	DT	CC	LRE
1 (RMSE)	491.34	465.87	614.90	463.94	343.93	488.01	461.44	343.19	484.45
3 (RMSE)	621.12	434.01	780.05	590.67	371.41	490.89	580.92	371.19	481.53
6_E (RMSE)	414.62	186.03	239.72	939.18	203.70	238.51	867.80	203.05	234.40
1 (MAE)	378.01	375.02	515.25	363.82	277.42	445.94	361.66	276.91	441.49
3 (MAE)	480.88	345.23	634.51	450.97	282.94	371.48	444.12	283.57	363.76
6_E (MAE)	311.01	234.93	188.08	681.15	156.53	174.89	639.10	157.07	176.95

**Table 12:** Results of the ARIMAX models under different transferability strategies for spatial transferability (200 m).

Transferability strategies	Cycleway 1			Cycleway 3		
	DT	CC	LRE	DT	CC	LRE
	Estimate (S.E.)	Estimate (S.E.)	Estimate (S.E.)	Estimate (S.E.)	Estimate (S.E.)	Estimate (S.E.)
Intercept	18.1650 (1.0839)	–	5.9401 (0.2938)	18.1650 (1.0839)	–	8.9245 (0.7763)
Post-intervention indicator	–	–		–	–	–
Time-since-intervention	− 0.0206 (0.0013)	− 0.0192 (0.0011)	− 0.0153 (0.0015)	− 0.0206 (0.0013)	− 0.0174 (0.0002)	− 0.0209 (0.0014)
Method to travel to work (bicycle)	–	− 0.1387 (0.0100)	0.2220 (0.0243)	–	− 0.1438 (0.0140)	− 0.0503 (0.0131)
Distance to work less than 2 km	0.1504 (0.0298)	0.0318 (0.0068)	–	0.1504 (0.0298)	–	–
Distance to work between 2–5 km	− 0.1405 (0.0499)	− 0.0442 (0.0046)	− 0.0348 (0.0070)	− 0.1405 (0.0499)	0.0385 (0.0073)	–
Female	–	− 0.0309 (0.0032)	− 0.0272 (0.0064)	–	− 0.0717 (0.0026)	− 0.0900 (0.0178)
Age between 20–49	− 0.1536 (0.0163)	− 0.0146 (0.0045)	–	− 0.1536 (0.0163)	− 0.0801 (0.0025)	–
Age between 50–74	− 0.1341 (0.0080)	0.0333 (0.0040)	–	− 0.1341 (0.0080)	− 0.1221 (0.0030)	–
Employed	0.0387 (0.0039)	–	− 0.0005 (0.0014)	0.0387 (0.0039)	0.0331 (0.0020)	–
Rented	− 0.0398 (0.0080)	0.0261 (0.0019)	–	− 0.0398 (0.0080)	− 0.0515 (0.0014)	0.00346 (0.0019)
Model statistics						
Log-likelihood	–	− 10336.29	− 6543.3	–	− 11429.01	− 6341.69
Akaike Information Criterion	–	20696.59	13106.6	–	22878.03	12703.37
Pseudo R-squared	–	0.18	0.14	–	0.11	0.13
Number of observations	4043	4043	4043	3732	3732	3732

“-” not statistically significant or not included.

**Table 13:** Results of the GAM model under different transferability strategies for spatial transferability (200 m).

Transferability strategies	Cycleway 1			Cycleway 3		
	DT	CC	LRE	DT	CC	LRE
	Estimate (S.E.)	Estimate (S.E.)	Estimate (S.E.)	Estimate (S.E.)	Estimate (S.E.)	Estimate (S.E.)
Intercept	8.0716 (0.1039)	–	3.6230 (0.0708)	8.0716 (0.1039)	–	4.6582 (0.0912)
Post-intervention indicator	0.2480 (0.0071)	0.1803 (0.0055)	0.1803 (0.0055)	0.2480 (0.0071)	0.6811 (0.0058)	0.1746 (0.0071)
Time-since-intervention	0.0572 (0.0012)	0.0672 (0.0009)	0.0672 (0.0009)	0.0572 (0.0012)	0.0401 (0.0010)	0.0671 (0.0012)
Method to travel to work (bicycle)	0.1367 (0.0018)	2.7939 (0.0174)	0.0289 (0.0009)	0.1367 (0.0018)	0.1679 (0.0023)	− 0.0405 (0.0002)
Distance to work less than 2 km	0.0883 (0.0012)	− 0.0250 (0.0007)	–	0.0883 (0.0012)	0.0183 (0.0004)	0.0101 (0.0009)
Distance to work between 2–5 km	− 0.2795 (0.0029)	− 1.1942 (0.0081)	0.0175 (0.0002)	− 0.2795 (0.0029)	0.0214 (0.0006)	–
Female	0.0879 (0.0023)	0.0630 (0.0011)	− 0.0495 (0.0002)	0.0879 (0.0023)	− 0.0043 (0.0001)	− 0.0933 (0.0002)
Age between 20–49	− 0.1332 (0.0008)	− 0.3393 (0.0021)	–	− 0.1332 (0.0008)	0.0431 (0.0007)	0.0148
Age between 50–74	− 0.0985 (0.0003)	− 0.5702 (0.0033)	–	− 0.0985 (0.0003)	0.0560 (0.0009)	–
Employed	0.0177 (0.0001)	0.4501 (0.0028)	− 0.0003 (0.0001)	0.0177 (0.0001)	0.0312 (0.0002)	–
Rented	− 0.0042 (0.0003)	0.0379 (0.0006)	–	− 0.0042 (0.0003)	− 0.0510 (0.0001)	0.0148 (0.0002)
Model statistics						
Log-likelihood	–	− 417284.4	− 448,793	–	− 281473.4	− 257821.8
Akaike Information Criterion	–	834606.8	897618.1	–	562984.9	515675.7
Adjusted *R*-squared	–	0.214	0.142	–	0.408	0.352
Number of observations	4043	4043	4043	3732	3732	3732

**Table 14:** Results of the GAMM+ARMA model under different transferability strategies for spatial transferability (200 m).

Transferability strategies	Cycleway 1			Cycleway 3		
	DT	CC	LRE	DT	CC	LRE
	Estimate (S.E.)	Estimate (S.E.)	Estimate (S.E.)	Estimate (S.E.)	Estimate (S.E.)	Estimate (S.E.)
Intercept	7.1409 (0.1964)	3.6512 (0.1297)	3.5239 (0.1438)	7.1409 (0.1964)	4.6677 (0.0920)	4.3951 (0.1767)
Post-intervention indicator	0.1897 (0.0105)	0.0851 (0.0064)	0.2324 (0.0081)	0.1897 (0.0105)	0.3916 (0.0058)	0.1938 (0.0105)
Time-since-intervention	0.0701 (0.0023)	0.0508 (0.0016)	0.0680 (0.0020)	0.0701 (0.0023)	0.0416 (0.0012)	0.0705 (0.0024)
Method to travel to work (bicycle)	0.1361 (0.0034)	− 0.0494 (0.0009)	0.0288 (0.0020)	0.1361 (0.0034)	0.0489 (0.0007)	− 0.0405 (0.0005)
Distance to work less than 2 km	0.0891 (0.0024)	0.0310 (0.0009)	–	0.0891 (0.0024)	0.0249 (0.0002)	0.0104 (0.0018)
Distance to work between 2–5 km	− 0.2784 (0.0056)	− 0.0395 (0.0005)	0.0176 (0.0005)	− 0.2784 (0.0056)	− 0.0052 (0.0003)	–
Female	0.0872 (0.0045)	− 0.0310 (0.0007)	− 0.0496 (0.0004)	0.0872 (0.0045)	0.0035 (0.0002)	− 0.0932 (0.0005)
Age between 20–49	− 0.1329 (0.0016)	− 0.0331 (0.0004)	–	− 0.1329 (0.0016)	− 0.0283 (0.0001)	–
Age between 50–74	− 0.0985 (0.0006)	− 0.0021 (0.0005)	–	− 0.0985 (0.0006)	− 0.0282 (0.0002)	–
Employed	0.0177 (0.0003)	0.0205 (0.0003)	− 0.0003 (0.0001)	0.0177 (0.0003)	0.0345 (0.0001)	–
Rented	− 0.0044 (0.0006)	0.0202 (0.0003)	−	− 0.0044 (0.0006)	− 0.0215 (0.0001)	0.0147 (0.0005)
Model statistics						
Log-likelihood	–	− 217690.5	− 165046.5	–	− 309085.1	− 102253.4
Akaike Information Criterion	–	435,411	330,113	–	618198.2	204526.7
Adjusted *R*-squared	–	0.284	0.139	–	0.384	0.349
Number of observations	4043	4043	4043	3732	3732	3732

**Table 15:** Results of the three modelling frameworks under different transferability strategies for temporal transferability (200 m).

Transferability strategies	ARIMAX			GAM			GAMM+ARMA		
	DT	CC	LRE	DT	CC	LRE	DT	CC	LRE
	Estimate	Estimate	Estimate	Estimate	Estimate	Estimate	Estimate	Estimate	Estimate
Intercept	5.7503 (0.2052)	–	5.8448 (0.1148)	5.3692 (0.0383)	3.8197 (0.0795)	3.8219 (0.0795)	5.3119 (0.0435)	3.7583 (0.0800)	3.7978 (0.0722)
Post-intervention indicator	0.3566 (0.1124)	− 0.1457 (0.0439)	–	0.0982 (0.0111)	0.3193 (0.0111)	0.3193 (0.0111)	0.1123 (0.0099)	0.3170 (0.0100)	0.3185 (0.0094)
Time-since-intervention	− 0.0175 (0.0072)	− 0.0145 (0.0021)	–	0.0996 (0.0077)	0.2331 (0.0090)	0.2331 (0.0090)	0.0992 (0.0075)	0.2409 (0.0086)	0.2352 (0.0077)
Method to travel to work (bicycle)	0.0560 (0.0114)	− 0.0305 (0.0059)	–	0.0240 (0.0003)	0.0013 (0.0003)	–	0.0223 (0.0011)	− 0.0046 (0.0014)	–
Distance to work less than 2 km	0.0230 (0.0071)	0.0186 (0.0035)	0.0176 (0.0033)	0.0194 (0.0002)	0.0143 (0.0002)	0.0143 (0.0002)	0.0173 (0.0007)	0.0110 (0.0008)	0.0104 (0.0008)
Distance to work between 2–5 km	− 0.1240 (0.0078)	− 0.0571 (0.0039)	− 0.0720 (0.0041)	− 0.0859 (0.0002)	− 0.0658 (0.0002)	− 0.0657 (0.0002)	− 0.0841 (0.0009)	− 0.0634 (0.0010)	− 0.0632 (0.0009)
Rented	0.0075 (0.0037)	0.0104 (0.0010)	0.0111 (0.0019)	0.0116 (0.0001)	0.0102 (0.0001)	0.0102 (0.0001)	0.0126 (0.0004)	0.0108 (0.0004)	0.0107 (0.0004)
Model statistics									
Log-likelihood	–	− 1719.31	− 1677.56	–	− 69328.47	− 69334.06	–	− 12,444	− 12827.84
Akaike information criterion	–	3452.62	3373.13	–	138688.9	138698.1	–	24,912	25675.68
Adjusted *R*-squared	–	0.12	0.16	–	0.41	0.41	–	0.408	0.407
Number of observations	1711	1711	1711	1711	1711	1711	1711	1711	1711

**Table 16:** RMSE and MAE of three modelling frameworks across transferability strategies and contexts (200 m).

Cycleway #	ARIMAX			GAM			GAMM+ARMA		
	DT	CC	LRE	DT	CC	LRE	DT	CC	LRE
1 (RMSE)	516.62	314.39	393.27	878.59	351.41	464.38	1613.44	325.39	464.36
3 (RMSE)	754.90	451.60	190.36	762.22	337.85	397.15	673.02	370.92	397.70
6_E (RMSE)	301.76	233.89	230.65	969.54	189.77	233.07	949.66	187.40	237.14
1 (MAE)	376.66	230.78	284.65	582.25	277.82	390.37	856.13	255.41	388.97
3 (MAE)	628.22	327.62	146.00	622.64	250.55	294.60	530.31	276.32	294.22
6_E (MAE)	225.60	185.93	183.80	688.98	152.12	183.22	678.40	150.51	186.69

**Table 17:** Results of the ARIMAX models under different transferability strategies for spatial transferability (400 m).

Transferability strategies	Cycleway 1			Cycleway 3		
	DT	CC	LRE	DT	CC	LRE
	Estimate (S.E.)	Estimate (S.E.)	Estimate (S.E.)	Estimate (S.E.)	Estimate (S.E.)	Estimate (S.E.)
Intercept	2.4340 (0.5915)	–	6.6087 (0.2837)	2.4340 (0.5915)	–	7.7252 (0.2051)
Post-intervention indicator	–	–	–	–	–	–
Time-since-intervention	− 0.0195 (0.0011)	− 0.0185 (0.0002)	− 0.0201 (0.0012)	− 0.0195 (0.0011)	− 0.0183 (0.0002)	− 0.0232 (0.0011)
Method to travel to work (bicycle)	0.0425 (0.0119)	− 0.1379 (0.0101)	–	0.0425 (0.0119)	–	0.0411 (0.0130)
Distance to work less than 2 km	− 0.0724 (0.0099)	− 0.0165 (0.0069)	–	− 0.0724 (0.0099)	0.0694 (0.0042)	–
Distance to work between 2–5 km	− 0.1045 (0.0125)	− 0.0643 (0.0046)	− 0.0201 (0.0041)	− 0.1045 (0.0125)	–	0.0376 (0.0054)
Female	0.0447 (0.0111)	0.0088 (0.0030)	− 0.0276 (0.0059)	0.0447 (0.0111)	0.0323 (0.0025)	− 0.0389 (0.0025)
Age between 20–49	–	–	–	–	− 0.0115 (0.0024)	–
Age between 50–74	0.0165 (0.0048)	0.0535 (0.0040)	–	0.0165 (0.0048)	0.0053 (0.0030)	–
Employed	− 0.0187 (0.0025)	− 0.0099 (0.0036)	− 0.0046 (0.0013)	− 0.0187 (0.0025)	0.0341 (0.0020)	–
Rented	0.0291 (0.0027)	0.0400 (0.0019)	–	0.0291 (0.0027)	− 0.0154 (0.0014)	− 0.0196 (0.0014)
Model statistics						
Log-likelihood	–	− 10331.09	− 10551.13	–	− 11272.73	− 11226.66
Akaike Information Criterion	–	20682.18	21122.26	–	22565.47	22473.32
Pseudo R-squared	–	0.18	0.12	–	0.15	0.16
Number of observations	6220	6220	6220	6864	6864	6864

“-” not statistically significant or not included.

**Table 18:** Results of the GAM model under different transferability strategies for spatial transferability (400 m).

Transferability strategies	Cycleway 1			Cycleway 3		
	DT	CC	LRE	DT	CC	LRE
	Estimate (S.E.)	Estimate (S.E.)	Estimate (S.E.)	Estimate (S.E.)	Estimate (S.E.)	Estimate (S.E.)
Intercept	− 0.9507 (0.0711)	2.7706 (0.0617)	3.7419 (0.0576)	− 0.9507 (0.0711)	4.7280 (0.0510)	3.6204 (0.0507)
Post-intervention indicator	0.3740 (0.0054)	0.1905 (0.0044)	0.1905 (0.0044)	0.3740 (0.0054)	0.5053 (0.0040)	0.4276 (0.0040)
Time-since-intervention	0.0591 (0.0009)	0.0615 (0.0008)	0.0615 (0.0008)	0.0591 (0.0009)	0.0397 (0.0007)	0.0630 (0.0007)
Method to travel to work (bicycle)	0.0556 (0.0004)	− 0.0493 (0.0003)	0.0547 (0.0002)	0.0556 (0.0004)	0.0490 (0.0004)	0.0944 (0.0003)
Distance to work less than 2 km	− 0.0459 (0.0003)	0.0309 (0.0004)	–	− 0.0459 (0.0003)	0.0250 (0.0001)	0.0037 (0.0001)
Distance to work between 2–5 km	− 0.1262 (0.0005)	− 0.0396 (0.0002)	− 0.0105 (0.0001)	− 0.1262 (0.0005)	− 0.0054 (0.0002)	–
Female	0.0405 (0.0004)	− 0.0309 (0.0003)	− 0.0425 (0.0001)	0.0405 (0.0004)	0.0036 (0.0001)	− 0.0240 (0.0001)
Age between 20–49	0.0092 (0.0001)	− 0.0330 (0.0001)	–	0.0092 (0.0001)	− 0.0283 (0.0001)	–
Age between 50–74	0.0250 (0.0002)	− 0.0021 (0.0002)	–	0.0250 (0.0002)	− 0.0282 (0.0001)	–
Employed	− 0.0214 (0.0001)	0.0205 (0.0001)	0.0003 (0.0001)	− 0.0214 (0.0001)	0.0345 (0.0001)	–
Rented	0.0325 (0.0001)	0.0202 (0.0001)	–	0.0325 (0.0001)	− 0.0214 (0.0001)	− 0.0138 (0.0001)
Model statistics						
Log-likelihood	–	− 615,858	− 691502.8	–	− 662977.1	− 844555.3
Akaike information criterion	–	1,231,756	1,383,038	–	1,325,994	1,689,143
Adjusted *R*-squared	–	0.29	0.204	–	0.386	0.201
Number of observations	6220	6220	6220	6864	6864	6864

**Table 19:** Results of the GAMM+ARMA model under different transferability strategies for spatial transferability (400 m).

Transferability strategies	Cycleway 1			Cycleway 3		
	DT	CC	LRE	DT	CC	LRE
	Estimate (S.E.)	Estimate (S.E.)	Estimate (S.E.)	Estimate (S.E.)	Estimate (S.E.)	Estimate (S.E.)
Intercept	− 2.3360 (0.1423)	3.6512 (0.1297)	3.6252 (0.1155)	− 2.3360 (0.1423)	4.6677 (0.0920)	3.1919 (0.1053)
Post-intervention indicator	0.2765 (0.0081)	0.0851 (0.0064)	0.2277 (0.0065)	0.2765 (0.0081)	0.3916 (0.0058)	0.2898 (0.0060)
Time-since-intervention	0.0784 (0.0019)	0.0508 (0.0016)	0.0627 (0.0016)	0.0784 (0.0019)	0.0416 (0.0012)	0.0703 (0.0014)
Method to travel to work (bicycle)	0.0556 (0.0008)	− 0.0494 (0.0009)	0.0546 (0.0004)	0.0556 (0.0008)	0.0489 (0.0007)	0.0943 (0.0008)
Distance to work less than 2 km	− 0.0460 (0.0008)	0.0310 (0.0009)	–	− 0.0460 (0.0008)	0.0249 (0.0002)	0.0037 (0.0002)
Distance to work between 2–5 km	− 0.1263 (0.0010)	− 0.0395 (0.0005)	− 0.0105 (0.0002)	− 0.1263 (0.0010)	− 0.0052 (0.0003)	–
Female	0.0407 (0.0008)	− 0.0310 (0.0007)	− 0.0425 (0.0003)	0.0407 (0.0008)	0.0035 (0.0002)	− 0.0240 (0.0001)
Age between 20–49	0.0092 (0.0003)	− 0.0331 (0.0004)	0.0003	0.0092 (0.0003)	− 0.0283 (0.0001)	–
Age between 50–74	0.0250 (0.0004)	− 0.0021 (0.0005)	–	0.0250 (0.0004)	− 0.0282 (0.0002)	–
Employed	− 0.0214 (0.0002)	0.0205 (0.0003)	0.0003 (0.0001)	− 0.0214 (0.0002)	0.0345 (0.0001)	–
Rented	0.0326 (0.0002)	0.0202 (0.0003)	–	0.0326 (0.0002)	− 0.0215 (0.0001)	− 0.0138 (0.0001)
Model statistics						
Log-likelihood	–	− 217690.5	− 255857.5	–	− 309085.1	− 334787.1
Akaike Information Criterion	–	435,411	511,735	–	618198.2	669596.1
Adjusted *R*-squared	–	0.284	0.201	–	0.384	0.196
Number of observations	6220	6220	6220	6864	6864	6864

**Table 20:** Results of the three modelling frameworks under different transferability strategies for temporal transferability (400 m).

Transferability strategies	ARIMAX			GAM			GAMM+ARMA		
	DT	CC	LRE	DT	CC	LRE	DT	CC	LRE
	Estimate	Estimate	Estimate	Estimate	Estimate	Estimate	Estimate	Estimate	Estimate
Intercept	5.0200 (0.2379)	−	5.3226 (0.0970)	5.0404 (0.0345)	3.7736 (0.0702)	3.7602 (0.0702)	5.0541 (0.0395)	3.7494 (0.0701)	3.7559 (0.0631)
Post-intervention indicator	0.2824 (0.1375)	− 0.1617 (0.0390)	–	0.0819 (0.0099)	0.2394 (0.0097)	0.2394 (0.0097)	0.0959 (0.0089)	0.2332 (0.0087)	0.2381 (0.0081)
Time-since-intervention	− 0.0154 (0.0088)	− 0.0136 (0.0019)	–	0.0776 (0.0069)	0.1920 (0.0079)	0.1920 (0.0079)	0.0781 (0.0069)	0.1981 (0.0075)	0.1944 (0.0067)
Method to travel to work (bicycle)	0.0860 (0.0154)	− 0.0375 (0.0056)	–	0.0277 (0.0003)	− 0.0066 (0.0003)	–	0.0248 (0.0011)	− 0.0125 (0.0014)	–
Distance to work less than 2 km	–	0.0083 (0.0031)	0.0066 (0.0030)	0.0080 (0.0002)	0.0045 (0.0002)	0.0046 (0.0002)	0.0078 (0.0006)	0.0018 (0.0007)	0.0012 (0.0007)
Distance to work between 2–5 km	− 0.0529 (0.0097)	− 0.0654 (0.0035)	− 0.0777 (0.0038)	− 0.0726 (0.0002)	− 0.0645 (0.0002)	− 0.0653 (0.0002)	− 0.0712 (0.0008)	− 0.0622 (0.0009)	− 0.0633 (0.0009)
Rented	–	0.0233 (0.0008)	0.0208 (0.0015)	0.0173 (0.0001)	0.0186 (0.0001)	0.0185 (0.0001)	0.0170 (0.0003)	0.0189 (0.0003)	0.0186 (0.0003)
Model statistics									
Log-likelihood	–	− 2057.04	− 2035.51	–	− 103793.2	− 103939.3	–	− 19938.82	− 20586.54
Akaike information criterion	–	4128.08	4089.03	–	207618.5	207908.5	–	39901.63	41193.08
Adjusted *R*-squared	–	0.15	0.17	–	0.305	0.304	–	0.305	0.304
Number of observations	2124	2124	2124	2124	2124	2124	2124	2124	2124

**Table 21:** RMSE and MAE of three modelling frameworks across transferability strategies and contexts (400 m).

Cycleway #	ARIMAX			GAM			GAMM+ARMA		
	DT	CC	LRE	DT	CC	LRE	DT	CC	LRE
1 (RMSE)	521.59	334.84	357.20	1366.96	326.74	496.03	1161.95	325.39	494.81
3 (RMSE)	682.20	406.78	430.27	798.50	372.13	568.44	784.09	370.92	569.76
6_E (RMSE)	346.34	292.71	295.86	801.52	210.06	258.97	779.83	208.82	258.35
1 (MAE)	386.94	244.36	269.87	778.44	257.55	416.03	713.99	255.41	413.91
3 (MAE)	526.06	279.85	305.68	639.51	276.99	425.92	631.97	276.32	426.53
6_E (MAE)	263.45	210.04	210.49	576.88	161.08	197.39	563.93	160.77	197.08
